# Targeting rapidly cycling receptors CD2 and CD7 increases nanoparticle delivery to primary CD4^+^ T cells

**DOI:** 10.1038/s41467-026-74981-2

**Published:** 2026-07-01

**Authors:** Paula M. Cevaal, Abdalla Ali, Marcel Doerflinger, Christina Cortez-Jugo, Abigail Tan, Haiyin Liu, Moore Z. Chen, Le Wang, Merle Dayton, Liana Mackiewicz, Stanislav Kan, Matthew Faria, Celine Gubser, René P. M. Lafleur, Robert De Rose, Angus P. R. Johnston, Frank Caruso, Michael Roche, Jori Symons, Sharon R. Lewin

**Affiliations:** 1 Department of Infectious Diseases, The University of Melbourne at The Peter Doherty Institute for Infection and Immunity, Melbourne, VIC Australia; 2 The Walter and Eliza Hall Institute of Medical Research, Melbourne, VIC Australia; 3 Department of Medical Biology, The University of Melbourne, Melbourne, VIC Australia; 4 Department of Chemical Engineering, The University of Melbourne, Parkville, VIC Australia; 5 Monash Institute of Pharmaceutical Sciences, Monash University, Melbourne, Australia; 6 Department of Biomedical Engineering, The University of Melbourne, Melbourne, VIC Australia; 7 Translational Virology, Department of Medical Microbiology, University Medical Center, Utrecht, the Netherlands; 8 Victorian Infectious Diseases Service, Royal Melbourne Hospital at The Peter Doherty Institute for Infection and Immunity, Melbourne, VIC Australia; 9 Department of Infectious Diseases, Alfred Hospital and Monash University, Melbourne, VIC Australia; 10 Present Address: Department of Biomedical Engineering, The University of Melbourne, Melbourne, VIC Australia; 11 Present Address: BioNTech SE, Mainz, Germany; 12 Present Address: Service of Infectious Disease, CHUV, Lausanne, Switzerland; 13 Present Address: Ardena, Oss, The Netherlands; 14 Present Address: Department of Infectious Diseases, The University of Melbourne at The Peter Doherty Institute for Infection and Immunity, Melbourne, VIC Australia

**Keywords:** Nanoparticles, Cell delivery, DNA and RNA

## Abstract

T cells are critically important to many diseases but are traditionally difficult to transfect. We hypothesise that the delivery of therapeutic cargo to T cells can be improved by targeting nanoparticles to surface receptors that undergo rapid receptor-mediated endocytosis. Using an internalisation assay that labelled intracellular and surface proteins with different fluorophores, we find that CD2 and CD7 exhibit significantly higher internalisation than other T cell receptors, such as CD3 or CD4. Targeting CD2 and CD7 improves nanoparticle internalisation by non-stimulated, primary CD4^+^ T cells and enhances the specificity of association to CD4^+^ T cells. Similarly, functionalising mRNA-lipid nanoparticles with antibodies targeting CD2 or CD7 enhances mRNA delivery to CD4^+^ T cells in vitro. Importantly, targeting CD2 or CD7 enables efficient lipid nanoparticle-mediated delivery of mRNA to T cells in blood and lymphoid tissue in vivo, demonstrating that targeting T cell receptor endocytosis can enhance nanoparticle-mediated drug delivery to T cells.

## Abstract (web summary)

T cells are important emerging therapeutics but are traditionally difficult to transfect. Here, the authors report on an optimised receptor targeting strategy to enhance delivery and internalisation of nanoparticles to human cells, demonstrating mRNA delivery in vitro and in vivo.

## Introduction

An ongoing challenge in the development of effective nanomedicines is delivering a sufficiently high dose of the therapeutic agent to intended cells in vivo^1^. T cells are recognised as being difficult to transfect due to their small size, high nucleus-to-cytoplasm ratio and low rates of endocytosis^2^. However, they are critically important in the adaptive immune response against viruses and cancers^3^ and they are subject to disease themselves, in the form of haematological malignancies or T cell-tropic viral infections^4–6^. HIV primarily infects activated and resting CD4^+^ T cells and integrates its genome into the host cell DNA. Following antiretroviral therapy (ART), the virus is rapidly cleared from plasma but in resting cells, HIV can become transcriptionally silent, establishing a latent infection that can persist life-long^7,8^. HIV can rebound from these cells as soon as ART is stopped. We therefore aimed to determine whether nanoparticle-based drug delivery could be targeted to CD4^+^ T cells, including resting CD4^+^ T cells, to enable more potent and more specific strategies to eliminate latently infected cells^2,9–12^.

Much effort to enhance cell-specific nanoparticle-based drug delivery has focused on decorating the surface of the nanoparticle with targeting moieties that specifically bind receptors on the target cell surface^13^, sometimes referred to as ‘active targeting’. Typically, the targeted receptor is chosen solely based on high expression levels and/or selective expression on the target cell^13^. This focus, however, overlooks the critical barriers that exist in certain hard-to-transfect cell types to nanoparticle internalisation and subsequent endosomal escape^2^. Indeed, a major barrier to successful nanoparticle delivery to T cells is their non-phagocytic nature and low rates of macropinocytosis in the absence of T cell activation (‘resting’ or dormant state)^14^. Clathrin-mediated endocytosis, in contrast, is used by resting cells to recycle cell-surface receptors^15^. While certain receptors are endocytosed continuously, the internalisation of others can be stimulated by binding specific ligands, though the degree of internalisation varies between receptors^16^.

We hypothesised that active targeting of nanoparticles to surface receptors with high internalisation kinetics could provide an avenue for enhancing uptake into T cells, including resting T cells. Using an in vitro assay to quantify receptor internalisation, we identified CD2 and CD7 as candidate receptors for active targeting approaches to resting T cells. Together, these results demonstrate a promising method for optimising the targeting efficiency to hard-to-transfect cell types.

## Results

### Selection of potential target receptors

Previous studies have focused on CD4 and CCR5 to enhance nanoparticle delivery to primary T-cells^17,18^. CD4 is the primary receptor and CCR5 is one of the co-receptors for HIV entry. CCR5, however, demonstrates poor selectivity towards T cells^19^ and CD4 has limited receptor internalisation^20,21^. We therefore identified a library of potential target receptors (Table 1) based on the following characteristics: (i) high expression on T cells, (ii) selective expression on T cells and not phagocytic cells, (iii) prior indication of high receptor cycling kinetics, (iv) associated with enrichment of latent HIV in people with HIV on ART and (v) prior usage for targeting nanoparticles to T cells.

**Table 1:** Overview of potential target receptors and their rationale for inclusion

Category	Marker	Rationale	Reference
General T cell markers	CD2	Pan-T cell marker; shown to enrich for HIV DNA in PWH on ART	^59,60^
	CD3	Pan-T cell marker; known to undergo receptor-mediated endocytosis and used previously to target nanoparticles to T cells	^39,61^
	CD4	HIV entry receptor; used previously to target nanoparticles to T cells	^17^
	CD5	Pan-T cell marker; used previously to target nanoparticles to T cells	
	CD7	Pan-T cell marker; used previously to target nanoparticles to T cells	^38,46^
Chemokine receptors	CXCR3	Shown to enrich for integrated HIV DNA in memory T cells of PWH on ART	
	CXCR4	HIV co-receptor	-
	CXCR5	Follicular T-helper cell (T_fh_) marker; T_fh_ are enriched for HIV DNA in PWH on ART	
	CXCR6	Primarily expressed in various T cell subsets	-
	CCR4	Primarily expressed in various T cell subsets	-
	CCR5	HIV co-receptor; used previously to target nanoparticles to T cells	
	CCR6	Shown to enrich for integrated HIV DNA in memory T cells of PWH on ART	^22,25^
	CCR7	Central memory T cell (T_cm_) marker; T_cm_ are enriched for HIV infection in PWH on ART	
	CCR9	Primarily expressed in lymphocytes	-
Immune checkpoint markers	OX40	Member of TNF receptor super family; shown to enrich for HIV in PWH on ART as part of a larger panel of markers	^27^
	CTLA-4	Immune checkpoint marker; shown to enrich for SIV in ART-treated rhesus macaques and HIV in ART-suppressed PWH, and known to undergo receptor-mediated endocytosis	^26,30,62^
	PD-1	Marker of immune exhaustion; shown to enrich for HIV in PWH both on and off ART	^31–35,62^
Cytokine receptor	CD25 / IL-2R	Activation marker; associated with HIV latency. Known to undergo receptor-mediated endocytosis upon stimulation, used previously to target nanoparticles to T cells	^27–29^

The resulting library included markers ubiquitously expressed on T cells, chemokine receptors, immune checkpoint receptors and activation markers. G protein-coupled chemokine receptors undergo receptor-mediated endocytosis following ligation and cells expressing certain chemokine receptors are enriched for latent HIV in people with HIV on ART^22–25^. Cells expressing immune checkpoint and activation markers are also enriched for latent HIV in people with HIV on ART^26–35^ and these receptors were therefore included. Receptors that were infrequently detected on total CD4^+^ T cells or had low receptor density, including CTLA-4, CXCR6 and CCR9, were excluded from further analysis (Supplementary Fig. 1).

### Receptor internalisation in T cells

**Fig. 1:**
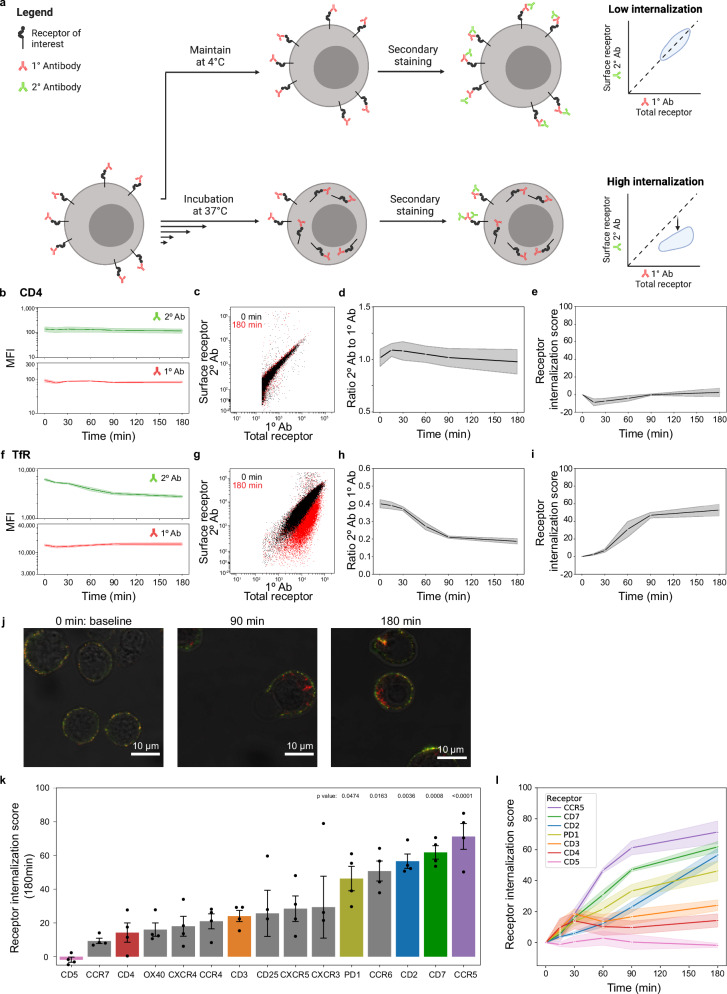
Receptor internalisation kinetics can be assessed using a dual-staining approach. **a** Schematic overview of the receptor internalisation assay. Created in BioRender. Cevaal, P. (https://BioRender.com/pogkzjt) **b**–**i** Representative results of the receptor internalisation assay in Jurkat T cells for a non-internalising (CD4, **b**–**e**) and strongly internalising (transferrin receptor, TfR, **f**–**i**) receptor as negative and positive controls, respectively. **b**, **f** Raw fluorescent signals (median fluorescent intensity, MFI) of primary and secondary antibody staining over time. The secondary antibody signal decreases over time in the presence of receptor internalisation, while the primary antibody signal remains constant irrespective of receptor internalisation kinetics. **c**, **g** Overlay of representative dot plots of the primary and secondary antibody signals at baseline (0 min, black) and 180 min (red), showing a downward shift over time in the fluorescence consistent with receptor internalisation. **d**, **h** Ratio of secondary antibody signal to primary antibody signal over time as a newly computed parameter per cell, quantifying any receptor internalisation. The baseline ratio at 0 min reflects the ratio of the respective degrees of labelling of the primary and secondary antibodies used. **e**, **i** Receptor internalisation score over time, calculated by normalising the values in (**d**) or (**h**) to the corresponding baseline (0 min timepoint). *n* = 3 independent repeats, values shown as mean ± SEM. **j** Representative confocal microscopy images of dual receptor staining at various timepoints. At baseline (0 min), no internalisation of the primary antibody-receptor complexes (red) is observed, and all receptors are dually stained with the secondary antibody (green). At later timepoints, primary antibody-receptor complexes can be observed within the cytoplasm, while the secondary antibody selectively co-stains receptors present on the cell surface. **k**, **l** Receptor internalisation score (IS) of receptors expressed on primary CD4^+^ T cells after 180 min (**k**) and change in IS over time for 7 receptors (**l**). *n* = 4 donors, values shown as mean ± SEM as either error bars (**k**) or shading (**l**). Significance was determined through one-way ANOVA and Dunnett’s multiple comparisons test using CD4 as reference.

To assess the ability of the receptors in our library to be internalised upon binding with a targeting antibody, we designed a high-throughput, flow cytometry-based internalisation assay based on previous work by Qureshi et al*.*^30^. We targeted CD71 (transferrin receptor, TfR – known to cycle extensively^36,37^) and CD4 (known to exhibit minimal endocytosis^20,21^) as positive and negative controls for receptor internalisation, respectively. We found that receptor internalisation could be tracked by staining a surface receptor of interest with a pH-stable, fluorescently labelled primary (1°) antibody (Fig. 1a, red) on ice, which is endocytosed along with the receptor during the subsequent incubation period at 37 °C. Any receptor-antibody complexes present on the cell surface after incubation could be co-stained with a fluorescently labelled anti-mouse IgG secondary (2°) antibody (Fig. 1a, green). Receptor internalisation resulted in a reduction of the secondary antibody signal over time and therefore, a reduced ratio of the secondary antibody signal to the primary antibody signal (Fig. 1a). Indeed, we observed negative and positive internalisation phenotypes for CD4 and TfR (Fig. 1b–d and Fig. 1f–h, respectively) and calculated a receptor internalisation score (IS, see Methods) of 2.5 ± 8.5 and 52.8 ± 6.8 (mean ± SEM) respectively after 180 min (Fig. 1e, i). Using confocal microscopy, we confirmed that the primary antibody signal could be internalised, whereas the secondary signal selectively co-stained receptor-antibody complexes that were present on the cell surface after incubation (Fig. 1j).

We next measured the receptor internalisation kinetics in Jurkat T cells and primary CD4^+^ T cells. While CD3 and CD5 receptor internalisation was high in Jurkat T cells, in total CD4^+^ T cells, CD5 receptor internalisation was not observed, and CD3 receptor internalisation was substantially reduced (Fig. 1k and Supplementary Fig. 2). In contrast, CD2 and CD7 internalisation was maintained in both Jurkat T cells and primary CD4^+^ T cells, with an IS of 56.6 ± 4.3 and 61.8 ± 4.0, respectively, in CD4^+^ T cells after 180 min. CCR5 was identified as a receptor with high internalisation kinetics with an IS of 71.3 ± 7.6 in primary T cells (Fig. 1k). CD7 and CCR5 were internalised more rapidly than CD2 (Fig. 1l). Interestingly, programmed cell death protein 1 (PD-1), an immune checkpoint marker shown to enrich for HIV in cells from people with HIV both on and off ART^31–35^, also showed high internalisation with an IS of 46.3 ± 7.2 after 180 min. CD2 and CD7, however, exhibited far greater expression and receptor density than CCR5 and PD-1 (Supplementary Fig. 1).

To confirm that the observed receptor internalisation also occurred in resting CD4^+^ T cells, which are most difficult to transfect^14^, we stained for the activation markers CD69 and HLA-DR in the receptor internalisation assay. On average, 94% of CD4^+^ T cells were found to be resting (CD69^_^/HLA-DR^_^) across all timepoints (Figure S3a). Though activated cells exhibited high receptor internalisation for both CD2 and CD7 (IS of 57.3 ± 4.3 and 64.5 ± 7.0 respectively), substantial internalisation was still observed in resting CD4^+^ T cells (IS of 56.5 ± 4.3 and 57.7 ± 4.4 respectively), as could be seen by a clear reduction in the ratio of 2° antibody signal over 1° antibody signal compared to baseline (0 min) (Supplementary Fig. 3b, c). Furthermore, receptor internalisation did not significantly differ between CD4^+^ T cells from people with HIV on suppressive ART and CD4^+^ T cells from HIV-negative donors (Supplementary Fig. 4).

Last, while the low and high internalisation observed for CD4 and CD7, respectively, corresponds closely to prior literature^20,21,38^, we wanted to assess the role of the chosen antibody clones in the observed receptor internalisation kinetics. For CD4, our prototype non-internalising receptor, we again observed a low level of receptor internalisation using two additional anti-CD4 antibody clones (Supplementary Fig. 5a). For CD2 and CD7, we observed receptor internalisation regardless of the antibody clone used, although differences in receptor internalisation kinetics were observed for some clones (Supplementary Fig. 5b, c). In particular, anti-CD7 antibody clone 6B7 exceeded the speed and total degree of receptor internalisation observed with the original anti-CD7 clone 848438. This difference could not be explained by differences in the induction of T cell activation markers between the anti-CD7 antibody clones (Supplementary Fig 5d–f). Combined, these data suggest that both the intrinsic target and the antibody clone can dictate internalisation.

### Receptor internalisation triggers nanoparticle uptake

**Fig. 2:**
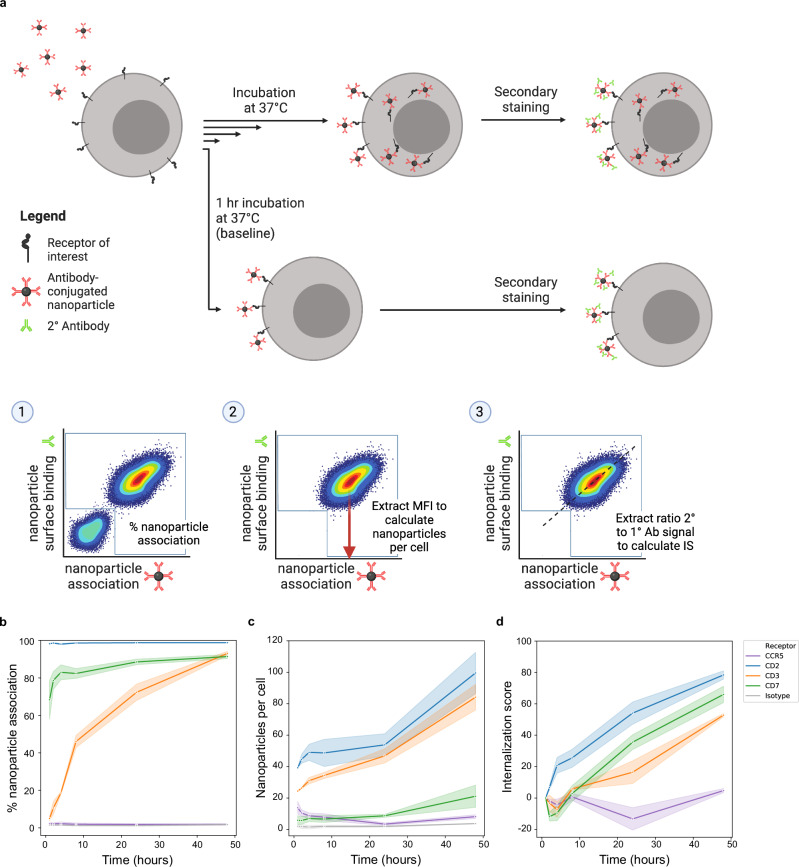
Dual staining of targeted nanoparticles allows for simultaneous assessment of association and internalisation. **a** Schematic overview of the nanoparticle internalisation assay, used to determine the percentage of cells associated with nanoparticles (1), the absolute number of nanoparticles associated per cell (2) and the nanoparticle internalisation score (3). Created in BioRender. Cevaal, P. (https://BioRender.com/pogkzjt) **b**–**d** Primary CD4^+^ T cells were incubated with nanoparticles targeting CCR5, CD2, CD3 or CD7, or untargeted (isotype) control particles and nanoparticle association and internalisation were assessed over time. **b** Percentage of live cells demonstrating association of each Ab@SPION over time. **c** Absolute number of each Ab@SPIONs associated per cell on cells found positive for nanoparticle association in (**b**). **d** Internalisation score of each Ab@SPIONs over time in cells found positive for nanoparticle association in (**b**), calculated by normalising the ratio of secondary to primary antibody signal to the first timepoint assessed (1 hr). An internalisation score for isotype@SPIONs could not reliably be determined due to the negligible percentage of cells found positive for nanoparticle association, and was therefore excluded from analysis. *n* = 4 donors, values shown as mean ± SEM.

We next examined whether the receptors with high internalisation kinetics could be exploited to enhance the internalisation of nanoparticles. To this end, we used 100 nm superparamagnetic iron oxide nanoparticles (SPIONs) coated in protein G for facile surface decoration with fluorescently labelled targeting antibodies (Ab@SPIONs) (Supplementary Fig 6a). Antibody-functionalisation did not change the nanoparticle hydrodynamic diameter, but the presence of targeting antibodies was confirmed using transmission electron microscopy (TEM) (Supplementary Fig 6b–d). We adapted the receptor internalisation assay to allow simultaneous assessment of the percentage of cells exhibiting nanoparticle association, the absolute number of nanoparticles associated as well as the degree of nanoparticle internalisation over time relative to the first baseline timepoint (Fig. 2a). In Jurkat T cells, targeting CD2 induced the highest as well as most rapid internalization of SPIONs into Jurkat T cells (IS of 85.7) while CD4 targeting resulted in a negligible IS of 0.42 after 48 hr (Supplementary Fig. 7). These findings are consistent with the internalization kinetics for these two receptors (Supplementary Fig. 2), demonstrating that targeting receptors with high internalization kinetics is a promising strategy for the rational design of delivery systems.

We therefore proceeded to assess Ab@SPIONs targeting CD2, CD7 and CCR5 – the receptors that exhibited the most potent receptor internalisation in primary CD4^+^ T cells (Fig. 2b–d and Figure S8). CD3@SPIONs were included as a control, as CD3 internalisation remains one of the most well-described examples of receptor-mediated endocytosis in primary T cells^39^. Targeting CD2 and CD7 resulted in nanoparticle association in 98% and 68% of CD4^+^ T cells respectively after 1 hr, which for CD7 increased to 91% at 48 hr (Fig. 2b). Targeting CD2 resulted in a higher dose of nanoparticles per cell (99.6 ± 14.4) compared to CD7 (21.1 ± 7.2) (Fig. 2c), despite both receptors exhibiting similar antibody binding capacities (Supplementary Fig 1). As the antibody binding capacity reflects the maximum binding in a saturating amount of antibody, these findings potentially reflect different affinities of the CD2 *versus* the CD7 antibody for their cognate receptors, resulting in reduced efficiency of CD7 nanoparticle association in a setting with a limited number of nanoparticles in culture. Nonetheless, both CD2 and CD7 triggered high internalisation of associated nanoparticles with IS scores of 78.4 ± 2.8 and 66.0 ± 5.1 for CD2 and CD7, respectively, exceeding the internalisation achieved by CD3 targeting (Fig. 2d). Nanoparticle internalisation of untargeted control nanoparticles could not reliably be determined due to the low percentage of CD4^+^ T cells that exhibited nanoparticle association (Fig. 2b) and was therefore excluded from further analysis.

Surprisingly, CCR5 targeting only resulted in nanoparticle association in 2.3% of CD4^+^ T cells (1 hr) compared to 1.5% for untargeted control particles (Fig. 2b), and a modest increase from 2.0 ± 0.86 to 13.8 ± 4.7 nanoparticles per cell at the 48 h timepoint (Fig. 2c). This low efficiency of T cell targeting using CCR5 is likely reflective of the low breadth of CCR5 expression in total CD4^+^ T cells (Supplementary Fig 1a), and the minimal increase in nanoparticles associated per cell may reflect the low receptor density in CCR5^+^/CD4^+^ T cells (Figure S1b). Furthermore, in those cells that associated with CCR5@SPIONs, minimal to no internalisation was observed (Fig. 2d). While the low levels of CCR5@SPION association may have compromised the detection of nanoparticle internalisation, these data suggest that CCR5 targeting is not a viable approach to enhance nanoparticle delivery to primary T cells.

Overall, these internalisation patterns corresponded closely to those observed in our receptor internalisation assay, suggesting that CD2 and CD7 are promising candidates for T cell targeting.

### Targeting enhances nanoparticle association with T cells in the presence of bystander cells

**Fig. 3:**
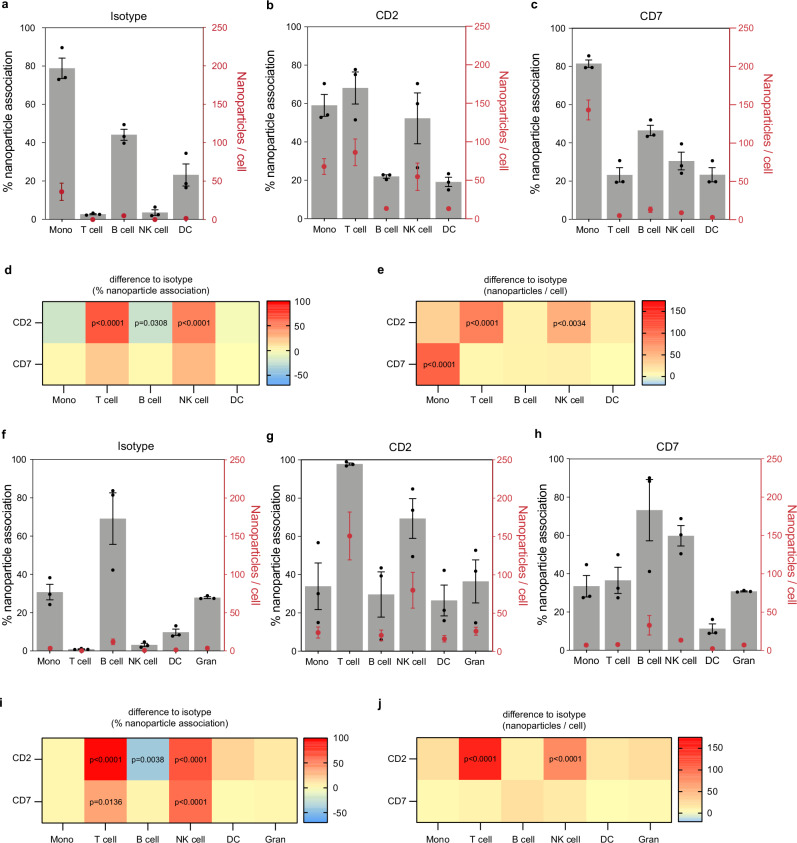
Receptor targeting can enhance nanoparticle association with T cells in the presence of bystander cells. **a**–**c** PBMC were incubated with untargeted control nanoparticles (isotype) (**a**) or nanoparticles targeted to CD2 (**b**) or CD7 (**c**) and nanoparticle-cell interactions with various PBMC subsets were assessed after 1 hr. For each nanoparticle, association was measured as the percentage of live cells that were associated with the Ab@SPION (bars, grey), as well as the absolute number of each Ab@SPIONs associated per cell on cells found positive for nanoparticle association (symbols, red). **d**,**e** Summary of data in (**a**–**c**), shown as a heat map with differences of means of each Ab@SPION compared to isotype control particles for each PBMC subset. **d** Difference in percentage nanoparticle association and; **e **difference in absolute number of Ab@SPIONs associated per cell. **f**–**j** Untargeted control nanoparticles (isotype) (**f**) or nanoparticles targeted to CD2 (**g**) or CD7 (**h**) were incubated with whole blood and nanoparticle-cell interactions with various leukocyte subsets were assessed after 1 hr. For each nanoparticle, association was measured as the percentage of live cells that demonstrate association of the Ab@SPION (bars, grey), as well as the absolute number of each Ab@SPIONs associated per cell of cells found positive for nanoparticle association (symbols, red). **i**, **j** Summary of data in (**f**–**h**), presented as differences of means of each Ab@SPION compared to untargeted control particles for each WBC subset. **i** Difference in percentage nanoparticle association; **j** difference in absolute number of Ab@SPIONs associated per cell. *n* = 4 donors, values shown as mean ± SEM. Significance was determined through one-way ANOVA and Bonferroni’s multiple comparisons test using untargeted control particles as reference. All other comparisons were non-significant. Mono monocyte; NK natural killer; DC dendritic cell; Gran granulocyte.

We next assessed whether targeting CD2 and CD7 could enhance nanoparticle association to T cells in peripheral blood mononuclear cells (PBMC). We demonstrated that CD2 and CD7 were expressed on T cells and natural killer (NK) cells with negligible expression in phagocytes (Figure S7). As expected, we found that untargeted control SPION particles exhibited strong association with phagocytic cells such as monocytes, B cells and DCs (79%, 44% and 23%, respectively), compared to 3% association with T cells (Fig. 3a–e). In stark contrast, targeting either CD2 or CD7 compared to isotype control particles resulted in increased SPION nanoparticle association with T cells (mean difference 65.4% association, *p* < 0.001 for CD2 and 20.5% association, *p* = 0.06 for CD7). The absolute number of nanoparticles per T cell was greatest upon CD2 targeting at 86 ± 17 (mean ± SEM) nanoparticles/cell.

When introduced into biological fluids, nanoparticles acquire a biomolecular corona that can change their physicochemical properties, which affects both targeting and stealth characteristics^40^. We therefore assessed the T cell targeting capabilities of the various Ab@SPIONs in the presence of blood serum (Supplementary Fig. 9), as described previously^41^. The distribution of nanoparticle association in the absence of targeting remained largely unchanged, with T cell association being practically absent, and monocytes and B cells accounting for the majority of nanoparticle association observed (Fig. 3f). CD2 targeting led to a dramatic increase in association with T cells, with 98% of T cells associating with an average of 151 ± 31 nanoparticles per cell (Fig. 3g). Compared to an untargeted control, CD2 targeting significantly reduced nanoparticle association to B cells (mean difference of −39.5% association, *p* = 0.0038), and significantly increased the percentage of association to NK cells (mean difference of 66.14% association, *p* < 0.0001) as well as the number of nanoparticles per NK cell (mean difference of 79.5 nanoparticles/cell, *p* < 0.0001). Overall, these effects were less pronounced than the targeting effect towards T cells (Fig. 3g, i, j).

The T cell targeting capacity of CD7@SPIONs compared to an untargeted control in whole blood showed a similar trend to CD2@SPIONs, significantly increasing the percentage of T cells associated with nanoparticles (mean difference of 35.6 ± 9.8% association, *p* = 0.0136). Despite CD2 and CD7 exhibiting similar receptor expression patterns in T cells, CD7@SPION association with T cells in whole blood reached only 37% (Fig. 3h). Compared to an untargeted control, CD7 targeting increased NK cell association (mean difference of 57.7 ± 9.8% association, *p* < 0.0001). These findings suggest that targeting CD2 and CD7 leads to pronounced association with T cells, and thus, potentially, internalisation in biologically relevant conditions.

### Targeting CD2 or CD7 enhances delivery of mRNA to T cells

**Fig. 4:**
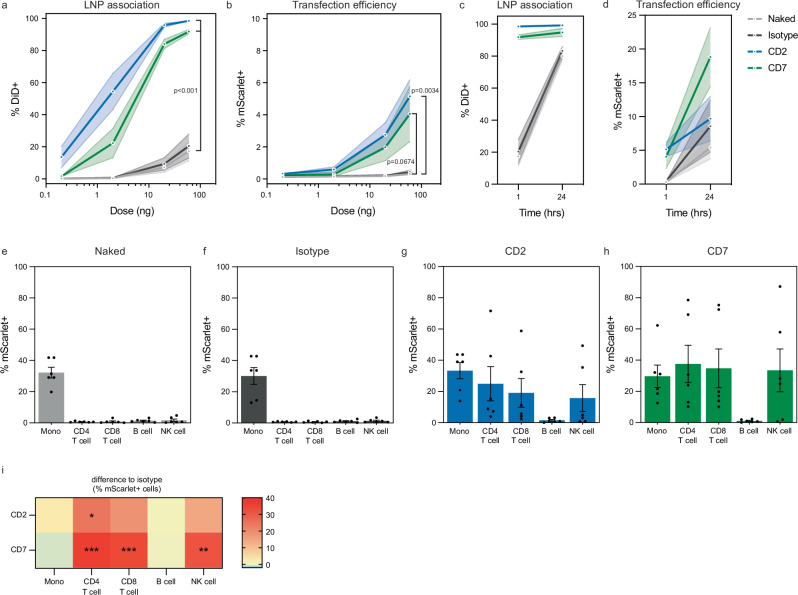
CD2- and CD7-targeting increases LNP-mediated delivery of functional mRNA to T cells. **a**, **b** Isolated CD4^+^ T cells were incubated with varying doses of DiD-labelled, mScarlet mRNA-LNPs targeted to CD2, CD7 or control LNPs for 1 hr, after which unbound LNPs were washed off. LNP association (**a**) and transfection efficiency (**b**) were determined after 24 hr. Significance was determined at the 60 ng dose using a one-way ANOVA compared to naked LNPs. **c**, **d** CD4^+^ T cells were continuously incubated with 60 ng DiD-labelled, mScarlet mRNA-LNPs targeted to CD2, CD7 or control LNPs for 24 hr and LNP association (**c**) and transfection efficiency (**d**) were compared to results from (**a**, **b**). *n* = 7 donors, values shown as mean ± SEM. **e**–**h** PBMCs were incubated with naked, non-functionalised, mScarlet mRNA-LNPs (200 ng mRNA, **e**), untargeted control LNPs (isotype, **f**) or LNPs targeted to CD2 (**g**) or CD7 (**h**) for 1 hr, after which unbound LNPs were washed off. The percentage of cells expressing mScarlet after 24 hours is shown. Expression in dendritic cells could not reliably be quantified due to low cell numbers. **i** Summary of data in (**f**–**h**), presented as differences in mean percentage mScarlet+ cells for each targeted LNP compared to isotype control LNP for each PBMC subset. *n* = 6 donors, values shown as mean ± SEM. Significance was determined through one-way ANOVA and Bonferroni’s multiple comparisons test using isotype control LNPs as reference. All other comparisons were non-significant. Mono, monocyte. * *p* ≤ 0.05, ** *p* ≤ 0.01, *** *p* ≤ 0.001.

We next determined whether targeting lipid nanoparticles (LNPs) to CD2 or CD7 could enhance delivery of an mScarlet reporter mRNA (Figure S10). Compared to isotype-targeted control LNPs, CD2- and CD7-targeted LNPs exhibited a 5.7- and 5.3-fold greater LNP association with primary CD4^+^ T cells, respectively (*p* < 0.001 for both receptors) (Fig. 4a). The increased LNP association coincided with an increased transfection efficiency for CD2 (16.3-fold increase, *p* = 0.0034) and to a lesser extent for CD7 (12.8-fold increase, *p* = 0.0674) (Fig. 4b). While the degree of association and transfection efficiency of untargeted control LNPs increased with increasing duration of LNP-T cell co-culture, the observed association of targeted LNPs occurred within the first hour of co-culture (Fig. 4c, d). These findings confirm an increased speed of nanoparticle association and internalisation in the presence of antibodies targeting CD2 and, to a lesser extent, CD7.

We then assessed the ability of CD2- or CD7-targeted LNPs to transfect T cells in the presence of bystander PBMCs. In the absence of targeting, mRNA was primarily delivered to and expressed in phagocytes, as expected, with minimal transfection of T cells ( < 1% mScarlet+ CD4^+^ T cells for both naked and isotype control LNPs) (Fig. 4e, f). In contrast, CD2- and CD7-targeting increased the transfection of T cells in the presence of PBMC to 24.9 ± 11% and 37.6 ± 12%, respectively. Functionalising the LNPs with targeting antibodies did not significantly alter the transfection efficiency of monocytes or B cells (Fig. 4g, h).

### Efficient and selective mRNA delivery to T cells in vivo

**Fig. 5:**
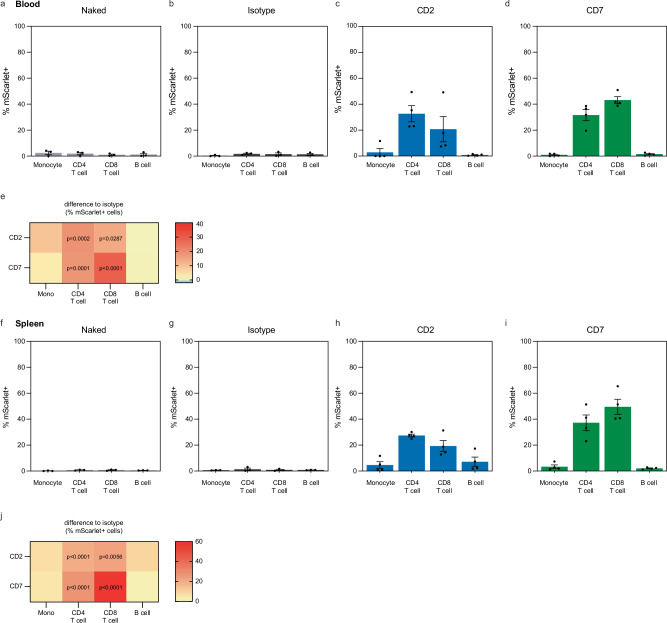
Targeting CD2 or CD7 enables mRNA delivery to T cells in vivo. Human immune system mice received a 10 µg dose of naked (**a**, **f**), isotype control (**b**, **g**), CD2-targeted (**c**, **h**) or CD7-targeted (**d**, **i**) mScarlet-LNP intravenously. After 16 h, the percentage mScarlet expression was determined in circulating (blood, **a**–**d**) or spleen-resident (spleen, **f**–**i**) human immune cell subsets. **e**, **j** Summary of data in **b**–**d** (**e**) or **g**–**i** (**j**), presented as differences in mean percentage mScarlet+ cells for each targeted LNP compared to isotype control LNP for each blood or spleen cell subset. *n* = 3 mice (naked, isotype) or *n* = 4 mice (CD2, CD7), values shown as mean ± SEM. Significance was determined through one-way ANOVA and Bonferroni’s multiple comparisons test using isotype control LNPs as reference. All other comparisons were non-significant. Mono, monocyte.

To validate whether CD2- or CD7-targeted LNPs enabled mRNA delivery to T cells in vivo, we employed a Human Immune System (HIS) mouse model based on NOD.Cg-Prkdc^scid^ Il2rg^tm1Wjl^/SzJ (NSG) mice reconstituted with human cord blood hematopoietic stem cells^42,43^. These mice have residual mouse myeloid, but no mouse lymphoid cells^43^. Antibodies targeting human CD2 or CD7 were conjugated to a modified LNP formulation optimised to exhibit high immune evasion (stealth) and prolonged circulation half-life^44^. The targeted LNPs, encapsulating an mScarlet reporter mRNA, were administered by i.v. injection. Consistent with the stealth characteristic of this LNP formulation, after 16 hour treatment with non-targeted LNPs, minimal mScarlet expression ( < 3%) was observed in all circulating or spleen-resident human immune cells (Fig. 5a, b, e, f and Supplementary Fig 11). Upon functionalisation of the LNPs with anti-CD2 antibodies, 33 ± 6.3% of circulating CD4^+^ T cells and 27 ± 1.1% of spleen-resident CD4^+^ T cells expressed mScarlet. CD7-targeted LNPs yielded 32 ± 4.2% and 37 ± 6.0% mScarlet expression in circulating and spleen-resident CD4^+^ T cells, respectively. CD8^+^ T cells were transfected at similar efficiencies to the CD4^+^ T cells. Expression of mScarlet was restricted to the T cell compartment with <5% of B cells and monocytes expressing mScarlet in both blood and spleen (Fig. 5c, d, g, h). mScarlet expression was also observed only in T cells within lymph-nodes after administration of CD2- or CD7-targeted LNPs, albeit at lower levels than in blood and spleen (Supplementary Fig 12a–e). In all sampled tissues, there was some expression of mScarlet in residual mouse-derived immune cells following the administration of CD2- or CD7-targeted LNPs, potentially indicating a degree of antibody cross-reactivity between mouse and human CD2 and CD7 antigens (Supplementary Fig 12f–h). Overall, these findings demonstrate that targeting CD2 or CD7 enables efficient and selective delivery of mRNA to T cells in vivo.

## Discussion

Successful delivery of a nanomedicine to a specific cell in vivo generally requires nanoparticle association as well as internalisation, however, these two processes are rarely differentiated. We describe evidence that targeting receptors that undergo extensive receptor cycling, such as CD2 and CD7, can be exploited to enhance nanoparticle internalisation in primary CD4^+^ T cells, a cell type generally considered difficult to transfect^45^. CD2 and CD7 receptor internalisation was maintained in CD4^+^ T cells from people with HIV, improving the clinical relevance of our findings. The effects of CD2- and CD7-targeting extended to lipid nanoparticles delivering functional cargo in vitro and, similarly, enabled potent mRNA delivery to T cells in vivo. Our findings add valuable quantitative and comparative data to previous reports of efficient T cell targeting using anti-CD7 antibodies or single-chain variable fragments (scFv)^38,46^. To our knowledge, no prior reports exist on the use of CD2 as a receptor target to enhance delivery to T cells.

Specifically targeting therapeutics to the HIV reservoir is complicated by the lack of a clear biomarker for latently infected cells^27,35^. Our findings demonstrate that targets such as CD4, CXCR4 and CCR5 as the HIV entry receptors, in fact resulted in poor nanoparticle association and/or internalisation in CD4^+^ T cells. CD4-targeted lipid nanoparticles have previously been shown to enhance mRNA delivery to CD4^+^ T cells in vitro and in vivo compared to untargeted control particles^17^. Yet, despite being specifically designed to target T cells, in this study less than 1% of particles added associated with T cells^17^, indicating a clear need for improved targeting approaches.

We employed a novel assay that simultaneously quantified nanoparticle association and the degree of internalisation. Several historic and recent ‘nanoparticle internalization assays’ have been described, which are typically based on quenching of membrane-bound nanoparticles^47–53^. These approaches, however, lack quenching specificity which therefore limits the capacity for multiplexing^47–49^, or use specific conjugation strategies that are not compatible with all nanoparticle types^50–53^. Furthermore, none of these described assays allow for concurrent assessment of nanoparticle association. Our assay was agnostic to the nanomaterial used, could be combined with phenotypic antibody staining, and could be integrated with recently developed methods to absolutely quantify nanoparticle association^54^, thereby contributing to improved metrics and better reporting in the field of nanomedicine that will accelerate translation and progress towards clinical applications^55,56^.

Some caveats to our assays need to be noted. First, the calculation of the nanoparticle internalisation score relied on the normalisation of internalisation to the earliest timepoint assessed at one hour. For targeted nanoparticles exhibiting rapid internalisation, this could lead to an underestimation of the internalisation. Second, the accuracy of the internalisation score was compromised in conditions where the level of nanoparticle association was low. Third, our assay was unable to discriminate nanoparticle recycling – *i.e*., internalisation followed by exocytosis without subsequent dissociation from the cell – from retention on the cell membrane. This distinction may not be important, as such rapidly recycled nanoparticles are unlikely to contribute to the delivery of a therapeutic cargo. Last, we found that for the rapidly-internalising receptor CD7, receptor internalisation was further enhanced using one particular antibody clone (6B7). As the target epitopes and relative binding affinities of each clone are unknown, it is plausible that antibody clones targeting different epitopes induce different receptor internalisation kinetics. The importance of the targeted receptor epitope therefore needs further study.

## Methods

### Antibodies

Primary antibodies targeting the various receptors of interest were all purchased as mouse-anti-human Alexa Fluor 647-conjugated monoclonal antibodies. Anti-CCR4 (1G1, 557863), anti-CCR6 (11A9, 560466), anti-CCR7 (150503, 560816), anti-CCR9 (112509, 557975), anti-CD3 (UCHT1, 557706), anti-CD4 (RPA-T4, 557707), anti-PD-1 (AH12.1, 560838) and anti-transferrin receptor (OKT9, 566724) antibodies were purchased from BD Biosciences. Anti-CD25 (BC96, 302618), anti-CD5 (UCHT2, 300616), anti-CTLA-4 (L3D10, 349919), anti-CXCR3 (G025H7, 353712), anti-CXCR5 (J252D4, 356906), anti-CXCR6 (K041E5, 356008) and anti-OX40 (OX40, 350018) antibodies were purchased from Biolegend. Anti-CCR5 (45529, FAB184R), anti-CD2 (299812, FAB18561R), anti-CD7 (848438, FAB7579R), anti-CXCR4 (44716, FAB172R) and isotype control (133303, IC0041R) antibodies were purchased from R&D Systems. To assess the effect of antibody clones on receptor internalisation, the following additional antibodies were purchased: anti-CD2 (RPA2.10, 300242, Biolegend and TS1/8, 208242, Biolegend), anti-CD4 (OCT4, 566681, BD Biosciences and SK3, 244636, Biolegend), anti-CD7 (4H9, 568094, BD Biosciences and 6B7, 343118, Biolegend). Primary antibodies that were stored in a buffer containing sodium azide were buffer-exchanged into phosphate-buffered saline (PBS) using Zeba™ Spin Desalting Columns, 7 K MWCO (89882, Thermo Fisher Scientific). The secondary, PE-conjugated goat-anti-mouse antibody (P-852) was purchased from Thermo Fisher Scientific.

### Cell culture

The human Jurkat T lymphocyte cell line (clone JE6.1) was cultured in RPMI 1640 medium containing 10% heat-inactivated foetal calf serum (FCS), supplemented with 100 U/mL penicillin, 100 µg/mL streptomycin and 2 mM L-glutamine (RF10) in a humidified 37 °C, 5% CO_2_ incubator. For primary cell experiments, peripheral blood mononuclear cells (PBMC) were isolated from buffy coats obtained from the Australian Red Cross Blood Service via Ficoll-Paque (GE Healthcare, Champaign, IL) density centrifugation. Blood samples from people living with HIV were collected through leukapheresis at the University of California, San Francisco (UCSF) (USA) with informed consent and under institutional guidelines. People living with HIV included in these studies were aged 18 years or older and on suppressive antiretroviral treatment (ART) (viral load <50 copies/mL) for >3 years. The use of these samples was approved by the Human Research Ethics Committees at the University of Melbourne and the Institutional Review Board at UCSF. CD4^+^ T cells were subsequently isolated through negative magnetic-activated cell sorting using a CD4^+^ T cell isolation kit (130-096-533, Miltenyi Biotec). PBMC and isolated total CD4^+^ T cells were frozen in FCS supplemented with 10% DMSO and stored in liquid nitrogen until use. Prior to usage, total CD4^+^ T cells were thawed and rested overnight in RF10 supplemented with 2 U/mL IL-2. Subsequent experimental incubations of total CD4^+^ T cells were performed in the continued presence of 2 U/mL IL-2.

### Receptor internalisation assay

To assess T cell surface receptor internalisation kinetics, a dual antibody staining approach was used based on prior work^30^. Jurkat T cells or primary total CD4^+^ T cells were harvested, counted and washed once in cold PBS with 1% FCS and 1 µM EDTA (FACS wash buffer) prior to staining with titrated volumes of the respective primary antibodies for 30 min, on ice, in the dark. Cells were kept on ice to inhibit any active endocytic processes during the binding of the primary antibodies. Next, excess primary antibody was washed off and cells were resuspended in cold culture media prior to incubation at 37 °C for 0, 15, 30, 60, 90 or 180 min. After incubation, cells were immediately placed back on ice and kept cold throughout subsequent handling to inhibit further receptor internalisation. A secondary antibody stain with PE-conjugated goat-anti-mouse antibody was then performed for 30 min, on ice, in the dark to dually stain any receptor remaining on the cell surface, while any internalised receptors remained singly stained. All cells were stained with LIVE/DEAD™ Fixable Aqua Dead Cell Stain (L34957, Thermo Fisher Scientific) to allow gating on live cells during subsequent analysis. In primary cell experiments, cells were subsequently incubated with unlabelled IgG from murine serum (I5381, Sigma) to block any free anti-mouse epitope binding sites of the goat-anti-mouse secondary antibody, followed by staining against the activation markers HLA-DR (347363, BD Biosciences) and CD69 (564364, BD Biosciences) with mouse-anti-human antibodies. Cells were then fixed in PBS with 1% paraformaldehyde (PFA) prior to flow cytometry analysis using an LSR Fortessa II (BD Biosciences) or microscopy analysis (Zeiss LSM780 Confocal). To assess the induction of T cell activation markers by the different anti-CD7 antibody clones, primary total CD4^+^ T cells were incubated with primary antibody as above and excess antibody removed. Cells were then incubated for 48 hr prior to staining against HLA-DR (347363, BD Biosciences), CD69 (564364, BD Biosciences) and CD25 (356108, Biolegend).

### Nanoparticle preparation

100 nm Absolute Mag™ Protein G Magnetic Particles (WHM-X035, Creative Diagnostics) (1 mg/mL, 1.3 × 10^11^ particles / mL) were functionalised with Alexa Fluor 647-labelled primary antibodies to generate fluorescently labelled, targeted nanoparticles (Ab@SPIONs). In brief, 10 µg of nanoparticles was mixed with 1 µg of primary antibody in 100 µL of 10 mM phosphate buffer (PB), pH 7.4, supplemented with 0.05 mg/mL bovine serum albumin (BSA), vortexed briefly to mix, then incubated for 30 min at room temperature. Excess unbound antibody was removed by immobilizing the nanoparticles using a magnet (EasyEights™ EasySep™ Magnet, Stemcell) and washing twice with 10 mM PB, pH 7.4, 0.5 mg/mL BSA, before resuspending in equal volumes of that same buffer to ensure equal nanoparticle concentration across all Ab@SPIONs. Antibody-functionalised nanoparticle solutions were then sonicated briefly using an ultrasonic bath. The hydrodynamic diameter and polydispersity index (PDI) of the resulting solutions, as measures of nanoparticle size and uniformity, respectively, were subsequently analysed using dynamic light scattering (DLS) using a Zetasizer (Nano-ZS, Malvern Analytical). Immediately prior to cell experiments, nanoparticle solutions were sonicated again to ensure a single-particle suspension. Antibody clones used correspond with those used in the receptor internalisation assays.

### Nanoparticle internalisation assay

Jurkat T cells or primary total CD4^+^ T cells were harvested, counted and resuspended in RF10. Cells were then plated at 1.25 × 10^6^ cells / mL in 200 µL in a round-bottom 96-well culture plate, after which nanoparticles conjugated with respective fluorescently labelled, primary antibodies were added at a nanoparticle-to-cell ratio of 100: 1. Cells were incubated for 24 or 48 hr at 37 °C. To allow for simultaneous harvesting and analysis, the remaining timepoints were started “in reverse”; at 8, 4, 2 and 1 hr before harvesting in separate wells. For primary CD4^+^ T cells, all timepoints were run using this reverse time course setup. After incubation, cells were immediately pelleted (400 rcf, 4 min) in a pre-cooled centrifuge (4 °C) and kept cold throughout subsequent handling to inhibit further nanoparticle internalisation. Unbound nanoparticles were washed off through three washes with cold PBS, followed by cell staining with LIVE/DEAD™ Fixable Aqua Dead Cell Stain and a secondary PE-conjugated goat-anti-mouse antibody as per previous. Cells were fixed in PBS with 1% paraformaldehyde (PFA) prior to flow cytometry analysis using an LSR Fortessa II (BD Biosciences).

### Nanoparticle association assays

The interaction of the various targeted nanoparticles with fresh whole blood cells and PBMC was studied as previously described^57^. Whole blood was collected from healthy volunteers after informed consent through microbleeds into NH sodium heparin collection tubes (454051, Greiner Bio-One). Fluorescently labelled, targeted nanoparticles were added at a ratio of 5 × 10^7^ particles per 200 µL of whole blood and incubated for 1 hr at 37 °C. Cells were then transferred to ice and kept cold throughout subsequent handling to inhibit further nanoparticle association. Red blood cells were lysed through incubation with 1X Pharm Lyse™ solution (BD Biosciences) for 15 min on ice, after which leukocytes were stained with LIVE/DEAD™ Fixable Blue Dead Cell Stain (L23105, Thermo Fisher Scientific) for 30 min on ice, in the dark, followed by surface staining with anti-CD56-AF488 (B159, BD Biosciences), anti-CD19-PE (HIB19, BD Biosciences), anti-CD66b-PE/Dazzle594 (G10FS, Biolegend), anti-HLA-DR-Pacific Blue (L243, Pharmingen), anti-CD3-Brilliant Violet 510 (UCHT1, BD Biosciences) and anti-CD14-Brilliant Violet 785 (M5E2, Biolegend) for 30 min on ice, in the dark. Cells were fixed in PBS with 1% paraformaldehyde (PFA) prior to flow cytometry analysis using an LSR Fortessa II (BD Biosciences). The following cell subsets were identified: granulocytes (CD66b^+^), T cells (CD66b^-^/CD3^+^), monocytes (CD66b^-^/CD14^+^), NK cells (CD66b^-^/CD3^-^/CD14^-^/CD56^+^), B cells (CD66b^-^/CD3^-^/CD14^-^/CD19^+^), dendritic cells (DC) ((CD66b^-^/CD3^-^/CD14^-^/CD56^-^/CD19^-^/HLA-DR^+^). PBMC were obtained as described above, thawed and plated at 4 × 10^5^ cells per well in 100 µL in flat-bottom 96-well culture plates. Subsequently, respective targeted nanoparticles were added at a nanoparticle-to-cell ratio of 100:1 and cells were incubated for 1 hr at 37 °C. Cells were then phenotyped and analysed as described above.

### Absolute quantitation of fluorescence

Conversion of median fluorescent intensity (MFI) values of Alexa Fluor 647 fluorescence into an absolute number of nanoparticles was performed as published previously^54^. In brief, the absolute fluorescent intensity per nanoparticle was determined using a microplate reader (FLUOstar Omega, BMG Labtech) (Table S1). The fluorescence of cell samples measured on flow cytometry was converted to absolute fluorescence using quantitation beads (Quantum™ MESF 647 beads, Bangs laboratories). The degree of labelling of each antibody was determined using fluorescence spectroscopy (Fluorolog 3, HORIBA) (Table S2). The antibody binding capacity of cells was determined by dividing the absolute fluorescence by the degree of labelling of each corresponding antibody.

### Lipid nanoparticles

Lipid nanoparticles (LNP) encapsulating mScarlet reporter mRNA were synthesised through microfluidic mixing using a NanoAssemblr Benchtop (Precision Nanosystems). In brief, for in vitro experiments, SM102 (Sapphire Bioscience), DSPC (Avanti Polar Lipids), ß-sitosterol (Sigma-Aldrich) and DMG-PEG2000 (Avanti Polar Lipids) were mixed at a molar ratio of 50:10:38.5:1.5, to which 0.2% DiD fluorescent dye (1,1’-dioctadecyl-3,3,3’,3’-tetramethylindodicarbocyanine, 4-chlorobenzenesulfonate salt, Invitrogen) was added to track LNP association to cells. Formulations were performed at a flow rate ratio of 1.8 (aqueous): 1 (organic) and N/P ratio of 6:1. For in vivo experiments, SM102, DSPC, cholesterol (Sigma) and DSPE-PEG2000 (Avanti Polar Lipids) were mixed at a molar ratio of 50:10:38.5:1.5 and formulations were performed at a flow rate ratio of 3 (aqueous): 1 (organic). LNP size and polydispersity were determined by nanoparticle tracking analysis (NanoSight NS300). RNA encapsulation efficiency and total RNA concentration of the LNP solution were determined using a modified RiboGreen assay. Throughout in vitro and in vivo experiments, mRNA-LNP dosing is based on the total mRNA concentration of the mRNA-LNP solution.

To functionalise LNPs with targeting antibodies, LNPs were purified from ethanol using dialysis (Slide-A-Lyzer G3 Dialysis Cassettes, 10K MWCO, Thermo Scientific). Nanobodies specific to the Fc domain of mouse IgG1 antibodies were then conjugated to DSPE-PEG2000 using strain-promoted azide−alkyne cycloaddition (SPAAC) as described previously^58^. Nanobody-DSPE-PEG2000 conjugates were post-inserted into the surface of the LNPs, resulting in a nanobody array with the Fc-binding site facing outward at a density of ~200 nanobodies per LNP^44^. Nanobody-functionalized LNPs were then purified using Amicon Ultra-4 centrifugal filters (100K MWCO), after which mouse anti-human antibodies targeting CD2 (clone RPA2.10, Biolegend), CD7 (clone 124-1D1, Thermo Fisher) or isotype control antibodies (clone P3.6.2.8.1, Thermo Fisher) were captured in upright orientation onto the nanobody-conjugated LNP surface. Targeting antibodies were added at a two-fold excess of nanobody to antibody to ensure efficient capture of all antibodies, resulting in an estimated density of ~100 antibodies per LNP^44^.

### Generation of Human Immune System (HIS) mice

As previously described^42^, human cord blood CD34^+^ hematopoietic stem cells (Lonza, 80–90% purity) were thawed, resuspended and cultured in 1 mL of X-VIVO-10 media (Lonza) supplemented with 2% human serum albumin, 100 ng/mL human TPO (Peprotech), 100 ng/mL human Flt3L (Peprotech) and 300 ng/mL human SCF (Peprotech) at 37 °C, 5% CO_2_ for 3 days. Cells were then counted and resuspended in PBS. Newborn NOD.Cg-Prkdc^scid^ Il2rg^tm1Wjl^/SzJ mouse pups (NSG; The Jackson Laboratory) were purchased from The Jackson Laboratory (USA) and were bred and maintained in the Specific Pathogen Free (SPF) Physical Containment Level 2 (PC2) Bioresources Facility at WEHI. Mice were visually checked daily and physically examined weekly. Mice were kept in individually ventilated cages (IVC; Tecniplast) at a 12/12-h light/dark cycle and provided standard rodent chow and sterile acidified water ad libitum. Mice were sub-lethally irradiated (150 cGy) between 24 and 48 h after birth, and followed by injection of 35 µL 1 × 10^5^ CD34^+^ HSCs into the temporal facial vein. Mandibular bleeds were performed at sixteen weeks of age to assess hematopoietic reconstitution by flow cytometry (Cytek Aurora) (Supplementary Data 1). All animal experiments were approved by Animal Ethics Committee (projects 2022.019) and Institutional Biosafety Committee (IBC) at the Walter and Eliza Hall Institute (WEHI).

### In vivo LNP treatment and analysis

HIS mice were injected intravenously with 10 µg targeted or control mScarlet-LNP. After 16 hours, mice were euthanised with blood, spleen and lymph nodes (salivary gland, axillary, cervical, mesenteric, brachial, pancreatic) collected. Whole blood samples were treated with ACK solution twice to lyse red blood cells. Spleen and lymph nodes are mashed through a 70 µm cell strainer into complete RPMI media to get single cell suspension. Splenocytes were treated with ACK solution once to lyse red blood cells. Cells from 200 µL blood, 20% of total cells from lymph nodes and 5% of total splenocytes were placed into a 96-well U bottom plate. Cells were then stained with Zombie UV viability dye (BioLegend) and anti-huCD45-BV786 (HI30), anti-huCD24-PacBlue (M5E2), anti-huCD19-BV510 (SJ25C1), anti-huCD3-PE/Cy7 (SK7), anti-huCD8-APC (RPA-T8), anti-huCD4-APC/H7 (RPA-T4) (all BD Biosciences) and anti-moCD45.1-FITC (A20-1, WEHI), in the presence of blocking reagent (Human BD Fc Block, Fc1 and Mouse BD Fc Block, 2.4G2, BD Biosciences) followed by flow cytometry (Cytek Aurora) (Figure S9).

### Analysis

Flow cytometry analysis, compensation and cell subset gating were performed using FCS Express 7 or FlowJo (V10). Further analysis including conversion of relative fluorescence into absolute number of nanoparticles per cell and graphing was performed in Python, using the Pandas, Numpy, Matplotlib, Sklearn and Seaborn packages. Confocal images were processed using ImageJ. Statistical analyses were performed in GraphPad Prism 10.

To calculate the receptor or nanoparticle internalisation scores, cells were gated on single cells, live cells, then cells expressing the receptor of interest (for calculation of receptor internalisation) or positive for nanoparticle association (for calculation of nanoparticle internalisation). Within cells found positive for nanoparticle association, the median fluorescent intensity (MFI) of the Ab@SPION signal (AF647) was then extracted and converted into a measure of the average absolute number of nanoparticles associated per cell. In FCS Express, a new parameter was then computed (“Ratio primary (1°) to secondary (2°) antibody”), which was defined as the MFI of surface receptor expression or nanoparticle surface binding divided by the MFI of total receptor expression or nanoparticle association. The median of this new parameter was then extracted and normalised to the first timepoint analysed (baseline) to calculate the receptor or nanoparticle internalisation scores using Eq. 1:

1 $${Internalization}\,{score}=1-\frac{{Ratio}\,{1^{\circ}} {to}\,2^{\circ} {antibody}\,}{{Baseline}\,{ratio}\,1^{\circ} {to}\,2^{\circ} {antibody}} * 100$$

### Reporting summary

Further information on research design is available in the Nature Portfolio Reporting Summary linked to this article.

## Supplementary information

Supplementary Information

Description of Additional Supplementary Files

Supplemtary Data 1

Reporting Summary

Transparent Peer Review file

## Source data

Source data

## Data Availability

Source data are provided for all experimental results presented in the main manuscript (Figs. 1–5) and supplementary information (Supplementary Figs 1–12). Source data are provided with this paper.
