# Supplementary material for: Targeting rapidly cycling receptors CD2 and CD7 increases nanoparticle delivery to primary CD4+ T cells
Source: Nat Commun. 2026 Jul 1;17:8204. doi: 10.1038/s41467-026-74981-2 (PMC13462104; doi:10.1038/s41467-026-74981-2)
Supplement: Supplementary file 1 [file 41467_2026_74981_MOESM1_ESM.pdf]

Supplementary Information

Supplementary Table 1. Fluorescent intensities of Ab@SPIONs.

Nanoparticle	Experiment			
	Jurkat T cells	CD4⁺ T cells	PBMC/WBC	
**TfR@SPION**	305	-	-	(MESF/nanoparticle)
**CD2@SPION**	41.8	41.7	32.1	(MESF/nanoparticle)
**CD3@SPION**	38.7	48.1	48.1	(MESF/nanoparticle)
**CD4@SPION**	122	-	-	(MESF/nanoparticle)
**CD5@SPION**	158	-	-	(MESF/nanoparticle)
**CD7@SPION**	105	233	97.6	(MESF/nanoparticle)
**CXCR4@SPION**	339	-	-	(MESF/nanoparticle)
**CCR5@SPION**	-	373	243	(MESF/nanoparticle)
**Isotype@SPION**	190	358	175	(MESF/nanoparticle)

CD, cluster of differentiation; CCR, C-C chemokine receptor; CXCR, C-X-C chemokine receptor; MESF, molecules of equivalent soluble fluorochrome; PBMC, peripheral blood mononuclear cells; SPION, superparamagnetic iron oxide nanoparticles; TfR, transferrin receptor; WBC, whole blood cells.

Supplementary Table 2. Degree of labelling (DOL) of fluorescent primary antibodies.

Antibody	Clone	DOL
**CCR4**	1G1	1.72
**CCR5**	45529	2.35
**CCR6**	11A9	15.0
**CCR7**	150503	5.20
**CCR9**	112509	1.69
**CD2**	299812	1.92
**CD25**	BC96	7.07
**CD3**	UCHT1	7.92
**CD4**	RPA-T4	12.3
**CD5**	UCHT2	5.94
**CD7**	848438	0.97
**CTLA4**	L3D10	5.43
**CXCR3**	G025H7	5.11
**CXCR4**	44716	1.35
**CXCR5**	J252D4	4.35
**CXCR6**	K041E5	4.11
**IgG2B**	133303	9.78
**OX40**	OX40	6.24
**PD1**	AH12.1	5.28
**TfR**	OKT9	18.1

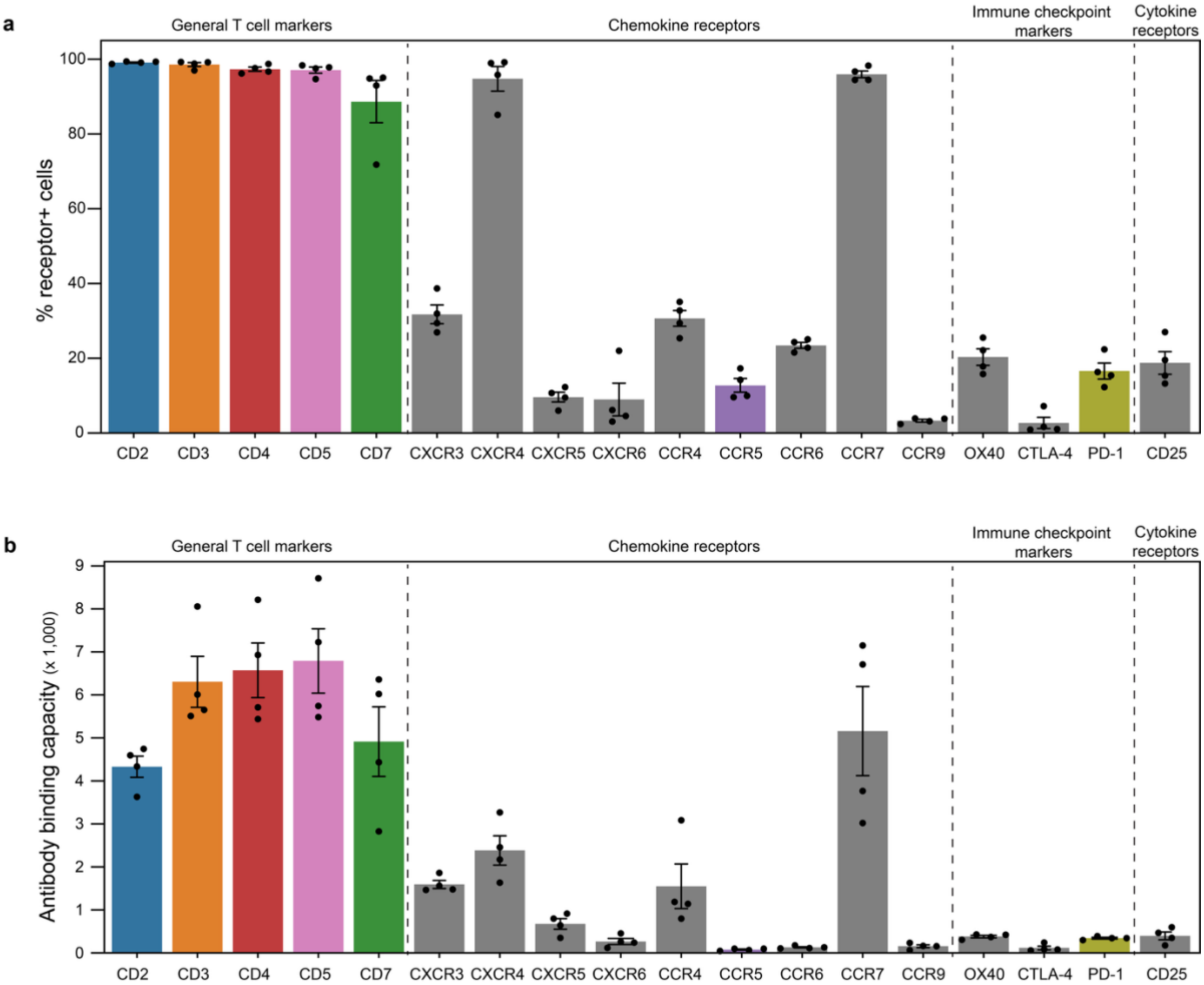

Supplementary Figure 1. Expression patterns on primary CD4⁺ T cells of surface receptors included in screening library. (a) Breadth of receptor expression measured by the percentage of live CD4⁺ T cells expressing the receptor of interest. (b) Receptor density on cells positive for the receptor is represented by the absolute number of anti-receptor antibodies bound per cell (antibody binding capacity). Values shown as mean ± SEM of n=4 donors.

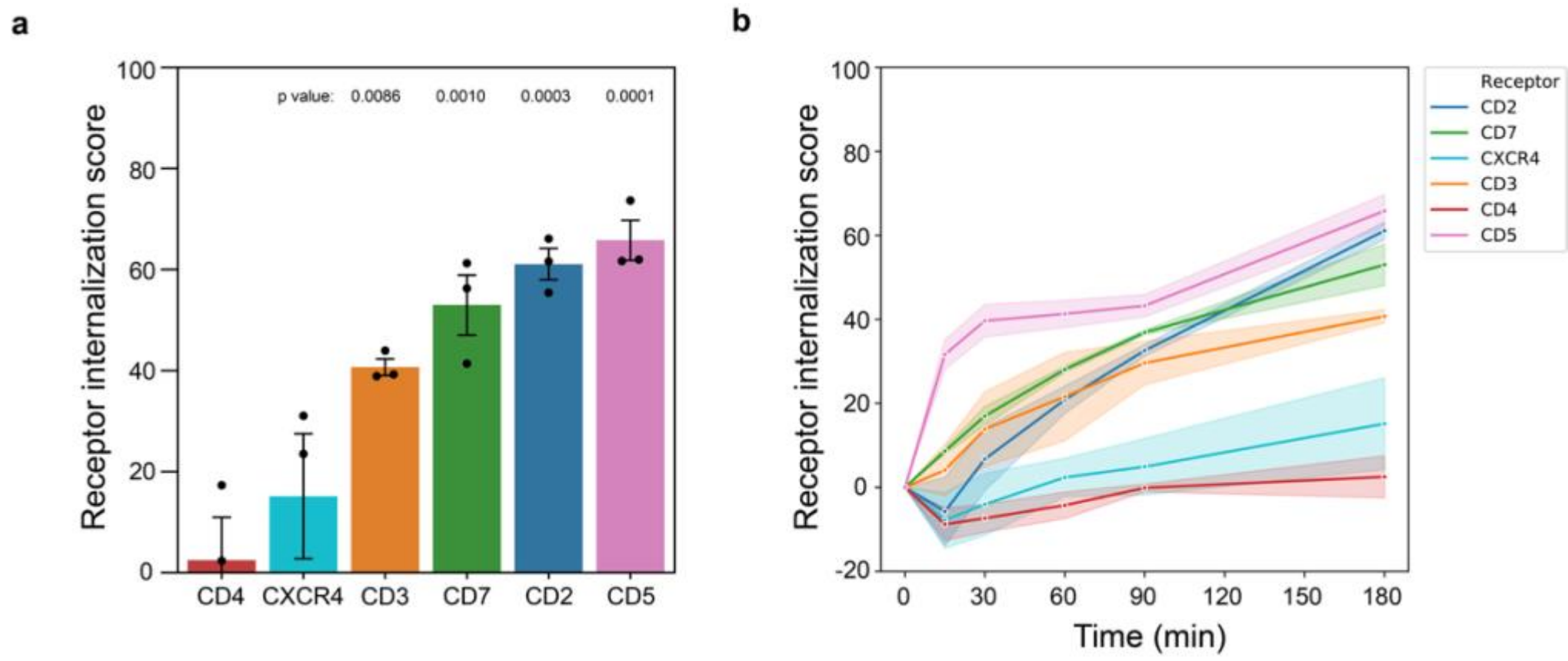

Supplementary Figure 2. Receptor internalisation kinetics in Jurkat T cells vary significantly. (a-b) Internalisation score of a selection of receptors expressed on Jurkat T cells after 180 min (a) and kinetics over time (b). n=3 independent repeats, values shown as mean ± SEM. SEM is shown as either error bars (a) or shading (b). Significance was determined through one-way ANOVA and Dunnett's multiple comparisons test using CD4 as reference.

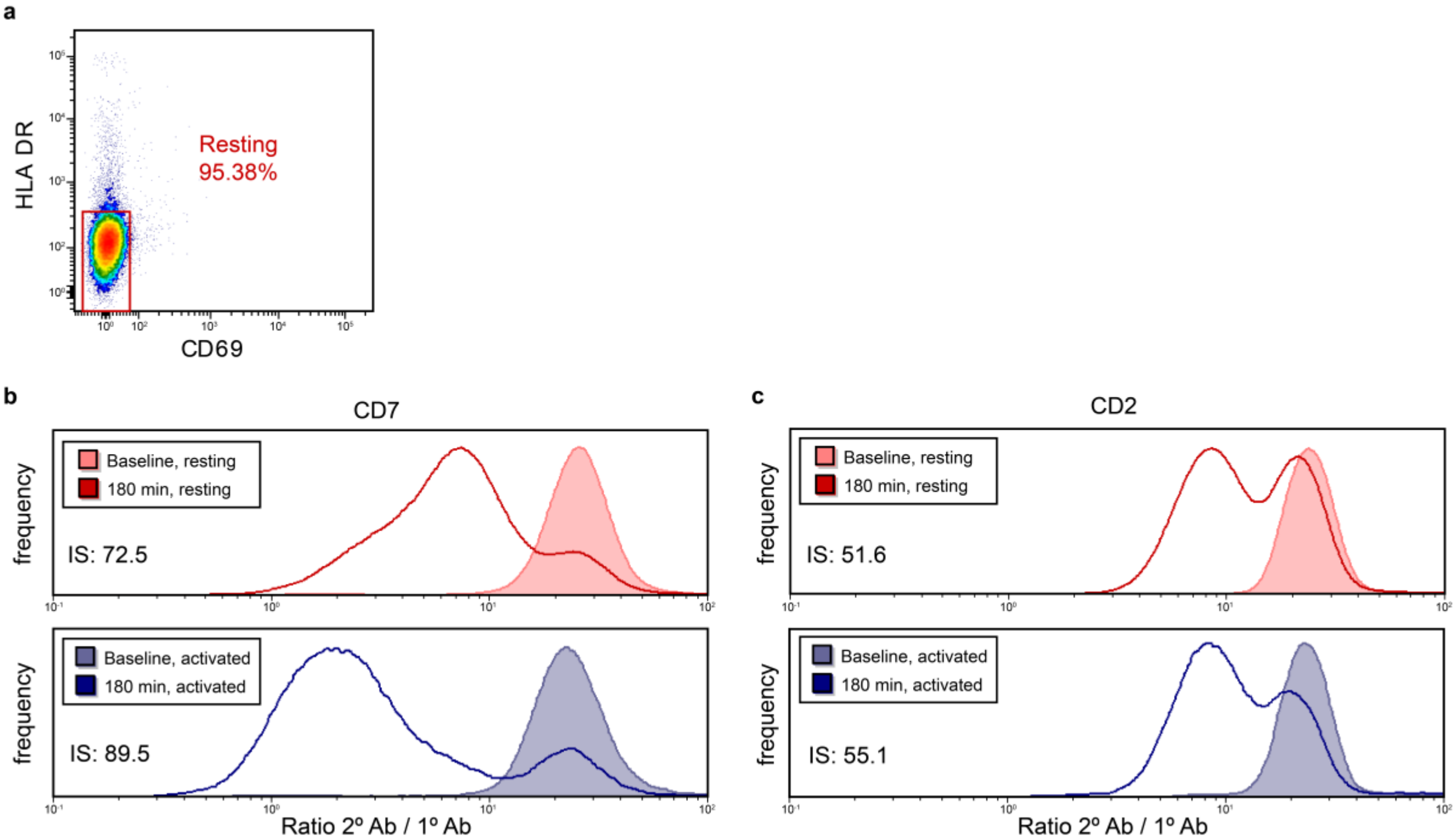

Supplementary Figure 3. Receptor internalisation occurs in both resting and activated CD4$^+$ T cells. Resting and activated CD4$^+$ T cells were identified by flow cytometry through staining for CD69 and HLA-DR after the dual staining procedure, to detect receptor internalisation at baseline and after 180 mins in both T cell populations. (a) Representative flow plot of activation marker expression within CD4$^+$ T cells. Double-negative cells were gated as resting, all others were defined as activated. (b,c) Representative histograms showing the distribution of the ratio of secondary (2°) to primary (1°) antibody (Ab) signals per cell, as a raw measure of CD7 (b) or CD2 (c) internalisation, for resting (top, red) and activated (bottom, blue) cells. Overlays depict the baseline ratio (filled curve) *versus* the ratio observed at the final timepoint of 180 min (line). Note that a lower ratio of secondary to primary antibody compared to baseline indicates receptor internalisation has occurred. Internalisation scores (IS) of the individual experimental replicates shown in the inset.

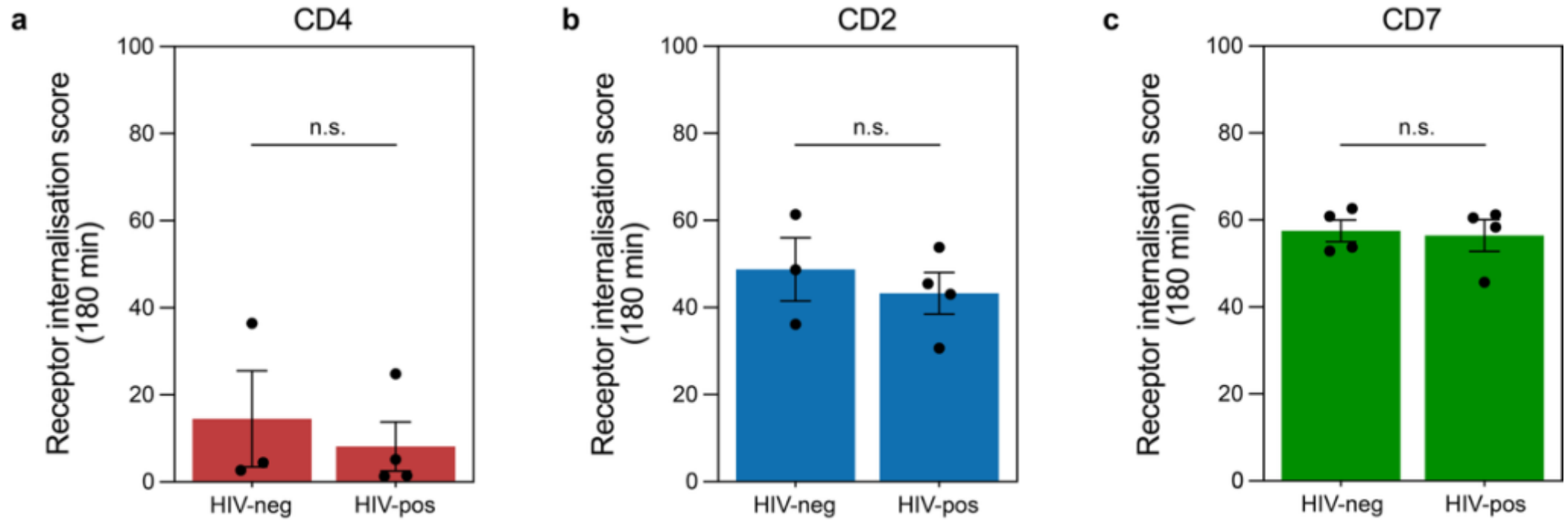

Supplementary Figure 4. Degrees of receptor internalisation are consistent between CD4$^+$ T cells from HIV-negative and -positive donors. Receptor internalisation score (IS) after 180 min of CD4 (a), CD2 (b) and CD7 (c) receptors expressed on primary CD4$^+$ T cells from HIV-negative donors or people living with HIV on suppressive antiretroviral treatment. n=3-4 donors, values shown as mean ± SEM. Significance was determined through two-way unpaired t-test; n.s. $p > 0.05$.

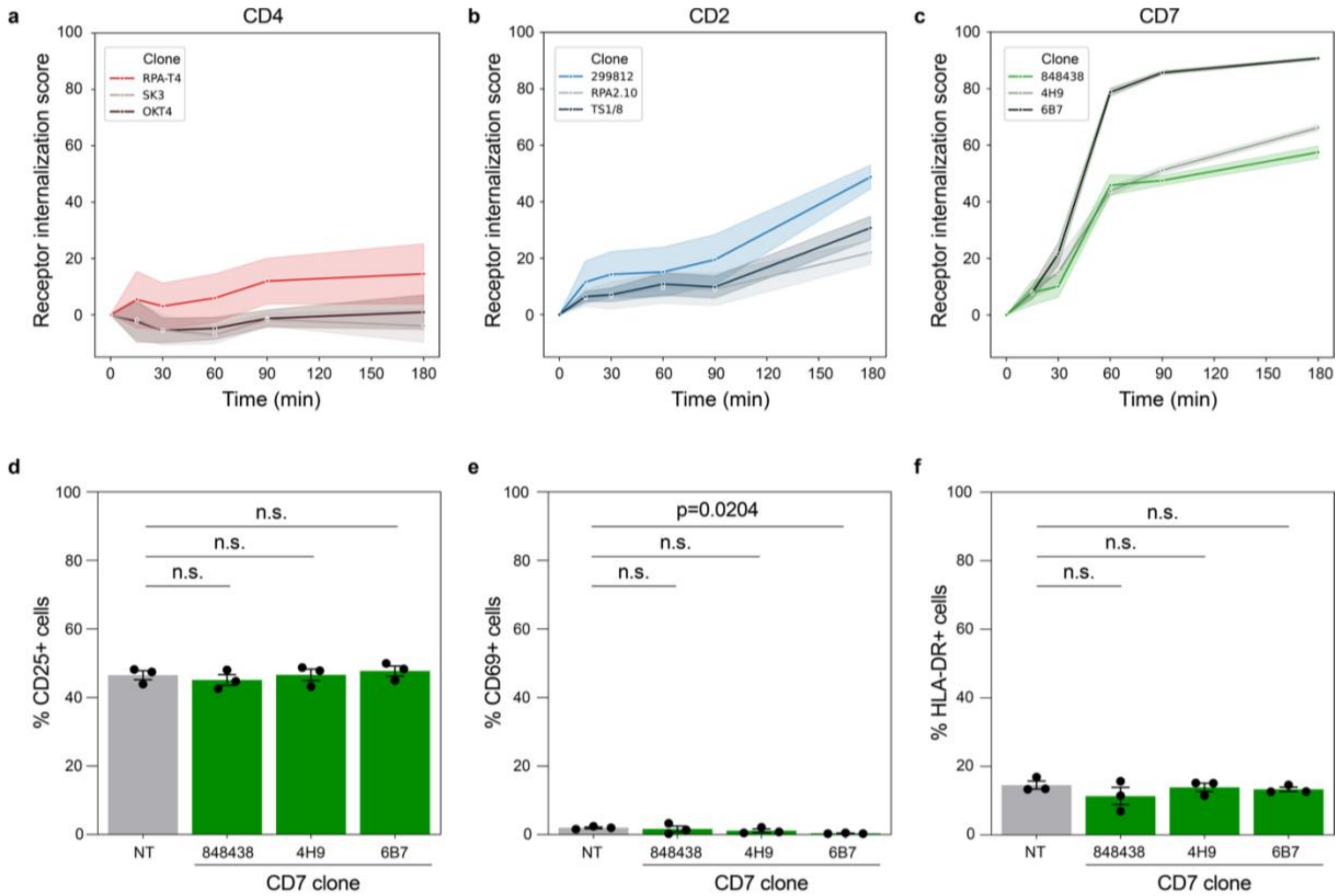

Supplementary Figure 5. Receptor internalisation may be enhanced by particular antibody clones. Receptor internalisation score (IS) over time of CD4 (a), CD2 (b) and CD7 (c) receptors expressed on primary $CD4^+$ T cells from HIV-negative donors. Original antibody clones depicted in red (CD4), blue (CD2) and green (CD7) shading; additional antibody clones against each receptor depicted in light and dark shades. n=4 donors, values shown as mean ± SEM. (d-f) Percentage of primary $CD4^+$ T cells expressing activation markers CD25 (d), CD69 (e) and HLA-DR (f) after 48 hr upon staining with original (848438) and additional anti-CD7 antibody clones (4H9 and 6B7), compared to non-treated (NT) control cells not incubated with any antibody. n=3 donors, values shown as mean ± SEM. Significance was determined through RM one-way ANOVA; n.s. p > 0.05.

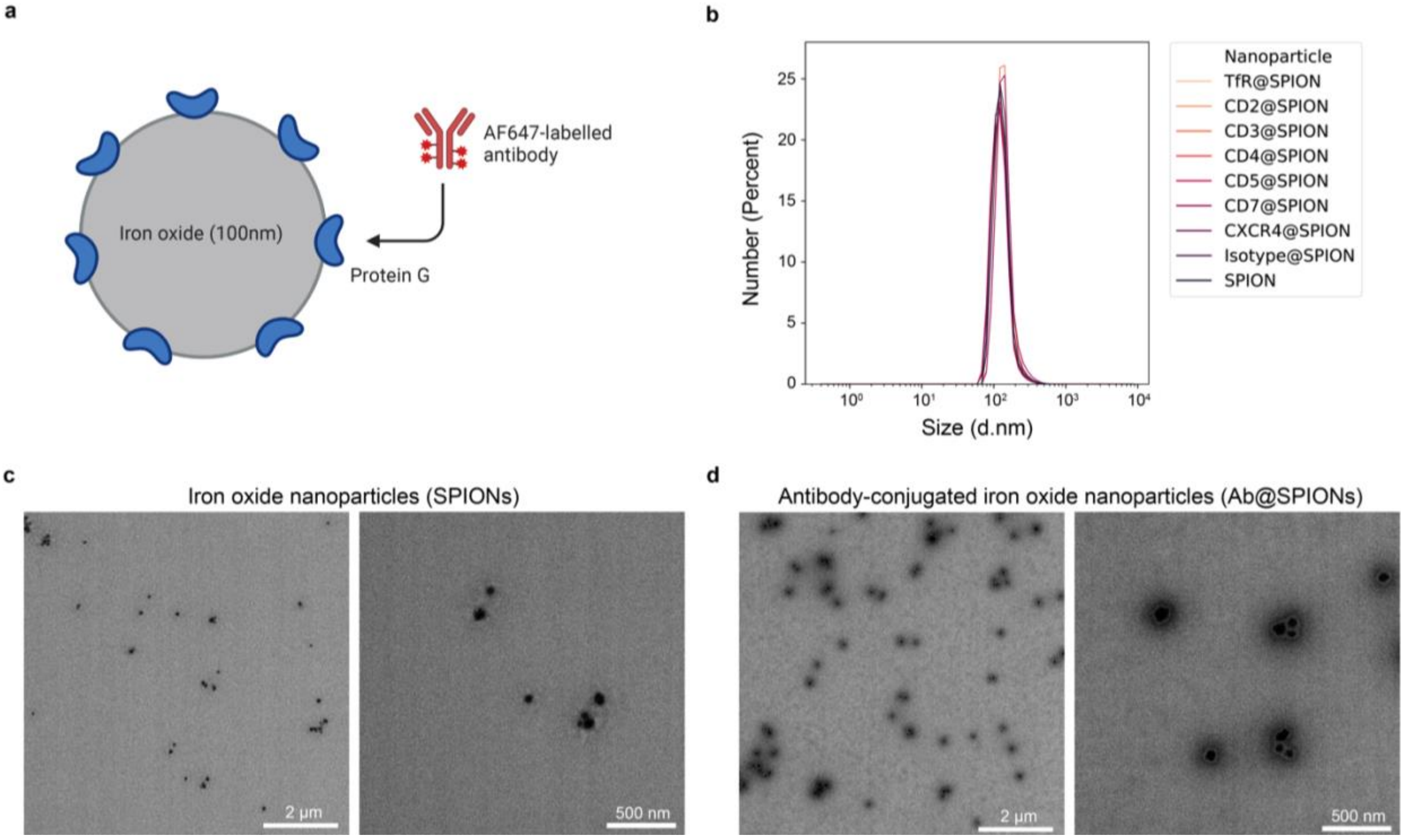

Supplementary Figure 6. Generation and characterisation of Ab@SPIONs. (a) 100 nm superparamagnetic iron oxide particles (SPIONs) coated in protein G were decorated with Alexa Fluor 647-labelled targeting antibodies through interaction between protein G and the Fc region of the antibodies. Excess unbound antibody was washed away and Ab@SPIONs were sonicated prior to use. Created in BioRender. Cevaal, P. (2026) https://BioRender.com/8epmlz2 (b) Size measurement of resulting Ab@SPIONs and unconjugated SPIONs as determined through dynamic light scattering. (c-d) Visualisation of antibody conjugation to SPIONs through transmission electron microscopy; the lighter area surrounding the SPIONS in (d) but not (c) indicates the addition of a protein layer.

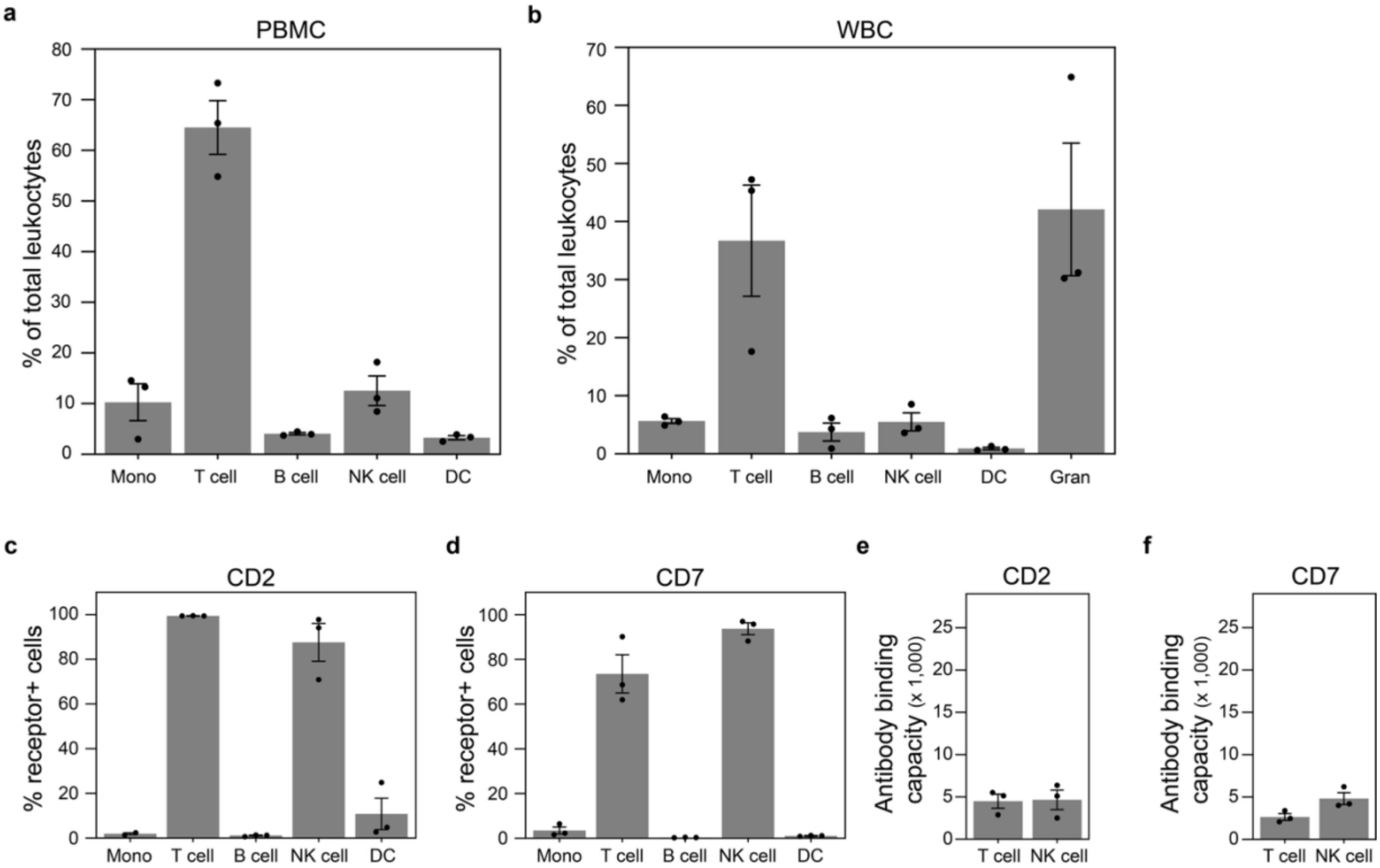

Supplementary Figure 7. Receptor targeting enhances nanoparticle association and internalisation in Jurkat T cells. (a) Breadth of receptor expression measured as the percentage of live Jurkat T cells expressing the receptor of interest. Values shown as mean ± SEM of n=3. (b) Receptor density on cells positive for receptor expression, represented by the absolute number of anti-receptor antibodies bound per cell (antibody binding capacity). For receptors not shown in this graph, no expression was detected. (c-e) Jurkat T cells were incubated with each of six targeting Ab@SPIONs or untargeted (isotype) control particles and nanoparticle association and internalisation were assessed over time. (c) Percentage of live cells demonstrating association of either Ab@SPION at each timepoint. (d) Absolute number of each Ab@SPIONs associated per cell of cells found positive for nanoparticle association in (c). (e) Internalisation score of each Ab@SPIONs over time in cells found positive for nanoparticle association in (c), calculated by normalising the ratio of secondary to primary antibody signal to the first timepoint assessed (1 hr). n=3 independent repeats, values shown as mean ± SEM.

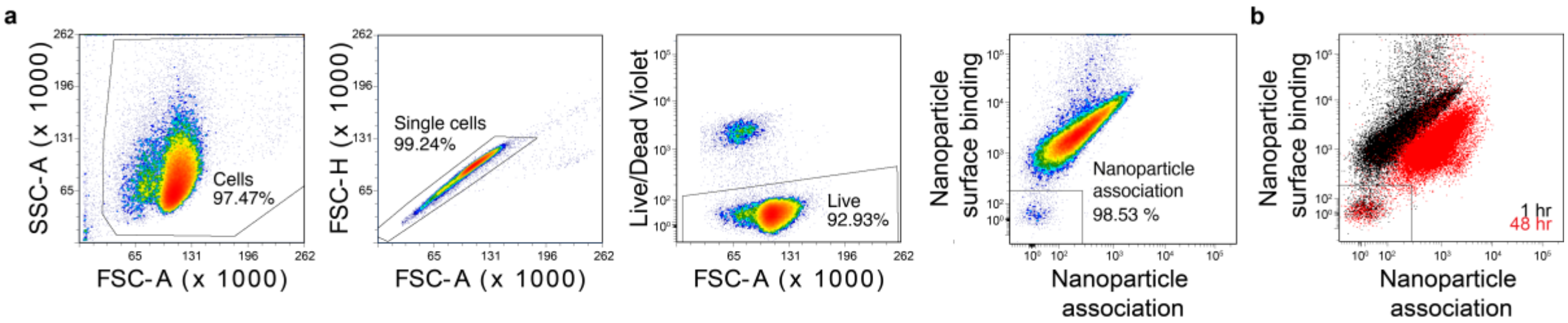

Supplementary Figure 8. Gating strategy for assessing nanoparticle association and internalisation in CD4$^+$ T cells. (a) Representative flow plots of CD4$^+$ T cells incubated with CD2@SPIONs for 1 hr, illustrating the gating strategy for quantifying nanoparticle association to live cells. (b) Overlay of representative flow plots of CD4$^+$ T cells incubated with CD2@SPIONs for 1 hr (black) or 48 hr (red), illustrating increased nanoparticle association over time combined with a reduced ratio of nanoparticle surface binding to nanoparticle association, indicating nanoparticle internalisation has occurred.

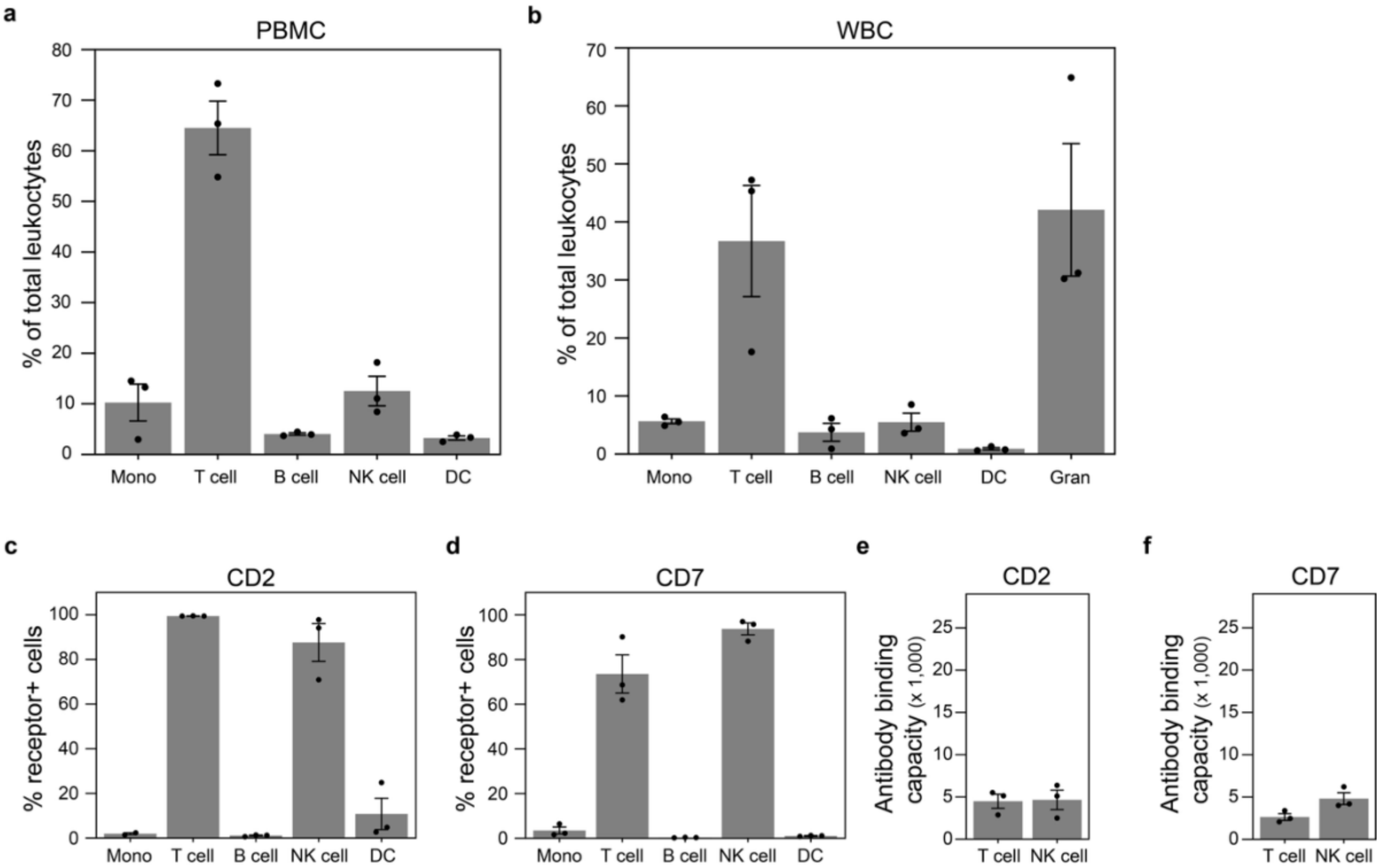

Supplementary Figure 9. Distribution of cell subsets and receptor expression levels in PBMC and whole blood. (a-b) In all nanoparticle association studies in PBMC (a) or whole blood (b), the proportion of monocytes, T cells, B cells, NK cells and dendritic cells of total leukocytes was determined. Cell subsets were identified as follows within single, live cells: granulocytes ($CD66b^+$), T cells ($CD66b^-/CD3^+$), monocytes ($CD66b^-/CD14^+$), NK cells ($CD66b^-/CD3^-/CD14^-/CD56^+$), B cells ($CD66b^-/CD3^-/CD14^-/CD19^+$), dendritic cells (DC) ($CD66b^-/CD3^-/CD14^-/CD56^-/CD19^-/HLA\text{-}DR^+$). (c-f) Expression of CD2 and CD7 was determined in the various PBMC subsets studied in association assays. (c-d) Breadth of receptor expression measured as the percentage of live cells expressing, CD2 (c) or CD7 (d). (e-f) Receptor density on cells positive for receptor expression, represented by the absolute number of anti-CD2 (e) or anti-CD7 (f) antibodies bound per cell (antibody binding capacity). Values shown as mean ± SEM of n=4 donors. Mono, monocyte; NK, natural killer; DC, dendritic cell.

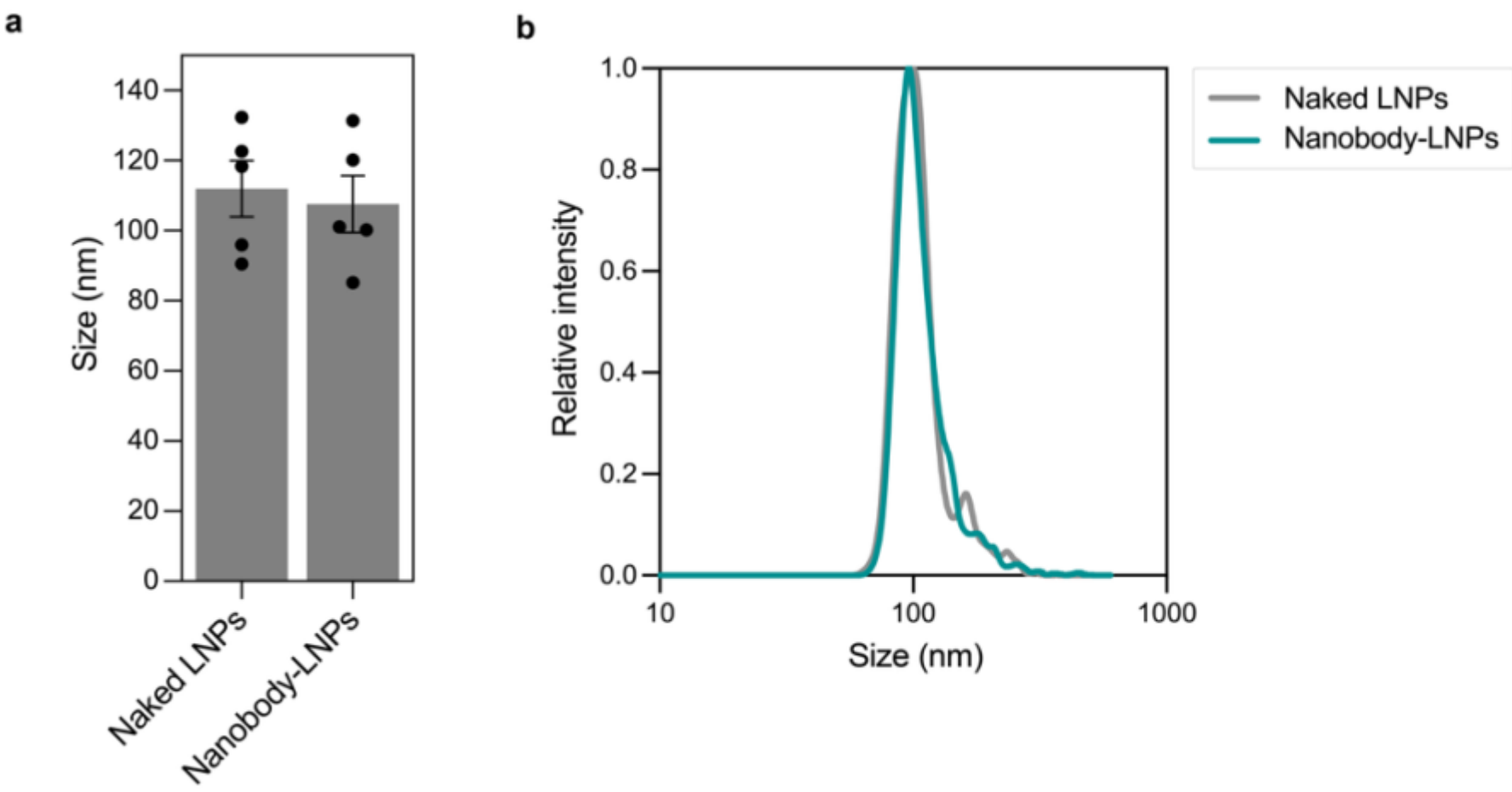

c

	EE
Naked LNPs	94.4%
Nanobody-LNPs	95.1%

Supplementary Figure 10. Characterisation of functionalised, mRNA-loaded lipid nanoparticles. (a,b) Size and size distribution of lipid nanoparticles (LNPs) were analysed by nanoparticle tracking analysis either immediately after formulation (naked LNPs), or after conjugation of the IgG1-capturing nanobody (nanobody-LNPs). (a) Size (nm) of n=5 LNP batches (mean ± SEM). (b) Representative size distribution of corresponding naked and nanobody-LNPs. (c) The mRNA encapsulation efficiency (EE) was determined using a modified RiboGreen assay for naked and nanobody-LNPs. Average EE for n=4 LNP batches each.

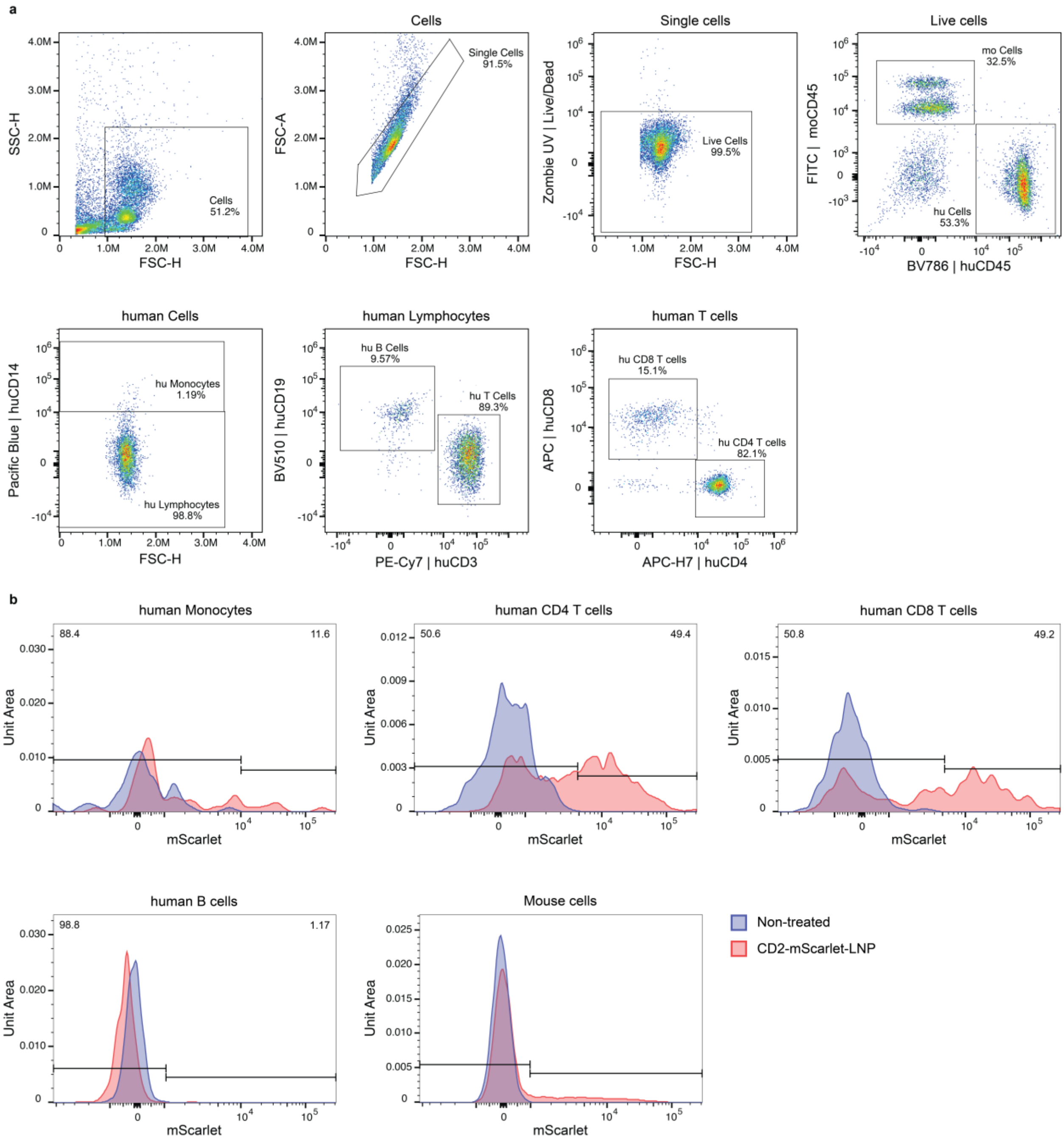

Supplementary Figure 11. Gating strategy used to identify mouse immune cells and human immune cell subsets expressing mScarlet in humanised mice treated with targeted or control mScarlet-LNP *in vivo*. (a) Representative flow plots of blood cells from a human immune system mouse treated with non-targeted (naked) mScarlet-LNPs *in vivo* to identify mouse immune cells (moCD45+), human monocytes (huCD45+/huCD14+), human B cells (huCD45+/huCD14-/huCD19+), human CD4 and CD8 T cells (huCD45+/huCD14-/huCD3+/huCD4+ or huCD8+). (b) Representative histograms of a human immune system mouse treated with non-targeted (naked) mScarlet-LNPs (blue) or CD2-mScarlet-LNP (red) illustrating the conservative gating strategy employed to classify cells expressing mScarlet as in Figures 5 and S10.

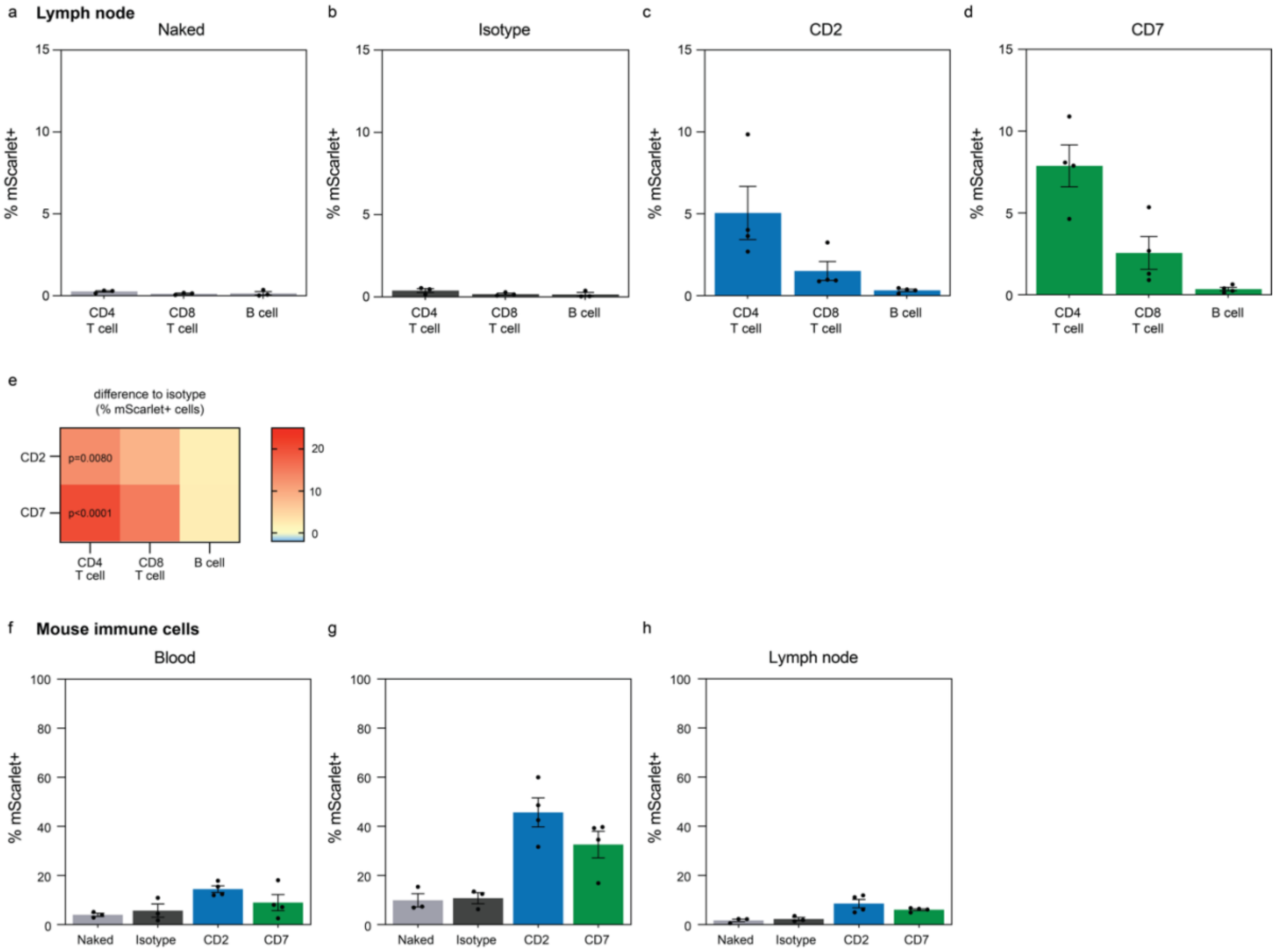

Supplementary Figure 12. mScarlet expression in human and mouse immune cells in blood, spleen and lymph node after *in vivo* mScarlet-LNP administration. (a-d) Human immune system mice received a 10 µg dose of naked (a), isotype control (b), CD2-targeted (c) or CD7-targeted (d) mScarlet-LNP intravenously as in Figure 5. After 16 hours, the percentage mScarlet expression was determined in lymph node-resident human lymphocyte subsets. (e) Summary of data in b-d, presented as differences in mean percentage mScarlet+ cells for each targeted LNP compared to isotype control LNP for each lymph node cell subset. Significance was determined through one-way ANOVA and Bonferroni's multiple comparisons test using isotype control LNPs as reference. All other comparisons were non-significant. (f-h) Simultaneously, the mScarlet expression was determined in residual mouse immune cells in blood (f), spleen (g) and lymph node (h) after administration of naked, isotype control, CD2- or CD7-targeted mScarlet LNPs. N=3 (naked, isotype) or n=4 (CD2, CD7), values shown as mean ± SEM.
