# Supplementary material for: Targeting rapidly cycling receptors CD2 and CD7 increases nanoparticle delivery to primary CD4+ T cells
Source: Nat Commun. 2026 Jul 1;17:8204. doi: 10.1038/s41467-026-74981-2 (PMC13462104; doi:10.1038/s41467-026-74981-2)
Supplement: Supplementary file 2 [file 41467_2026_74981_MOESM2_ESM.pdf]

Descriptions of Additional Supplementary Files

Supplementary Data 1: Reconstitution statistics of human immune system (HIS) mice. Percentage of mouse (mo) cells versus various human (hu) immune cell subsets (monocytes, B cells, CD4 T cells, CD8 T cells) (reconstitution) in blood, spleen and lymph node (LN) of human immune system (HIS) mice prior to treatment with CD2-targeted, CD7-targeted, isotype control or non-targeted control (naked) mScarlet-LNPs as in Figure 5 of the main manuscript. One control mouse that did not receive any LNPs (non-treated) was included for comparison. Treatment group, mouse age at day of sacrifice post-LNP treatment and mouse sex are indicated per individual.
