# Supplementary material for: Targeting rapidly cycling receptors CD2 and CD7 increases nanoparticle delivery to primary CD4+ T cells
Source: Nat Commun. 2026 Jul 1;17:8204. doi: 10.1038/s41467-026-74981-2 (PMC13462104; doi:10.1038/s41467-026-74981-2)
Supplement: Supplementary file 4 [file 41467_2026_74981_MOESM5_ESM.pdf]

nature portfolio

Peer Review File

Targeting rapidly cycling receptors CD2 and CD7 increases nanoparticle delivery to primary CD4+ T cells

Corresponding Author: Professor Sharon Lewin

This manuscript has been previously reviewed at another journal. This document only contains information relating to versions considered at Nature Communications.

This file contains all reviewer reports in order by version, followed by all author rebuttals in order by version.

Version 0:

Reviewer comments:

Reviewer #1

(Remarks to the Author)
The delivery of therapeutic cargo to resting primary T cells via nanoparticles poses significant challenges due to their inherently low transfection efficiency. In this study, Cevaal et al. suggest that targeting surface receptors with rapid internalization kinetics may enhance nanoparticle uptake and, consequently, improve transfection efficiency. Through in vitro internalization assay, the authors identified CD2 and CD7 as T cell surface markers with notably high internalization rates. They demonstrate that lipid nanoparticles (LNPs) functionalized with antibodies against CD2 or CD7 facilitate efficient mRNA delivery to primary T cells both in vitro and in vivo, utilizing humanized mouse models. The data presented are compelling, offering a promising strategy to address key challenges in T cell targeting. This work signifies a meaningful advancement in nanoparticle-mediated therapeutic delivery to primary T cells. However, the study also highlights several limitations, which are discussed below:

1. The intrinsic recycling rate of a surface marker can be significantly influenced by the binding epitope and affinity of the antibody that induces internalization. The current findings are derived from a single antibody clone chosen by the authors, raising questions about the generalizability of their conclusions to other antibody clones that target the same marker but possess different epitopes and affinities. For instance, although the authors assert that endocytosis for CD4 and CD5 is minimal, previous research has demonstrated that lipid nanoparticles (LNPs) functionalized with anti-CD4 or anti-CD5 antibodies can effectively mediate mRNA delivery to primary T cells in vivo. At the very least, for markers like CD2 and CD7, the authors should evaluate results across multiple antibody clones with clearly documented epitope specificity and binding affinities.

2. The authors demonstrated that the internalization kinetics of the same surface markers, such as CD3 and CD5, vary significantly between Jurkat cells and primary $CD4^+$ T cells. This variation underscores the importance of cellular context in determining the internalization kinetics of these markers. To enhance the clinical relevance of these findings, it is essential to compare the internalization kinetics of CD2 and CD7 in primary $CD4^+$ T cells derived from healthy donors with those from patients.

3. Characterization of the density of CD2 and CD7 antibodies on the functionalized, mRNA-loaded lipid nanoparticles is missing.

4. Since the in vitro receptor internalization assays for CD2 and CD7 were conducted in $CD4^+$ T cells rather than $CD8^+$ T cells, it would be more accurate to revise the title by replacing “primary T cells” with “primary $CD4^+$ T cells.”

5. Previous studies have shown that LNPs functionalized with anti-CD3 or anti-CD5 antibodies can effectively deliver mRNA to primary T cells in vivo, which seems to contrast with the conclusions presented in this manuscript. The authors should address these discrepancies in the discussion by comparing their findings with earlier research and providing possible explanations for the observed inconsistencies.

Version 1:

Reviewer comments:

Reviewer #1

(Remarks to the Author)
Although some questions are addressed, but there are a couple of concerns with the data provided. For question #1, the authors followed our suggestions and tested three different antibody clones. There are two concerns with the data (fig. S5)

1. In the original report, aCD7 (clone 84838) showed an internalization score of 61.8 ± 4.0 in $CD4^+$ T cells after 180 minutes (Fig.1 K-l). However, in this subsequent repeat experiment comparing three different aCD7 clones, the same clone (84838) exhibited an internalization score of less than 20 at the same time point (Fig.S5). This substantial inter-experimental variability (≈62 vs <20) warrants further explanations.

2. The internalization score of the aCD7 clone 6B7 is approximately twofold higher than that of clone 4H9 and fourfold higher than that of the original clone 84838 (Fig.S5). This magnitude of clone-dependent variability raises the question of whether nanoparticle delivery efficiency to $CD4^+$ T cells is driven primarily by intrinsic CD7 receptor internalization kinetics or by the specific antibody clone used for targeting. Additional evidence is needed to distinguish between these two possibilities in order to endorse the authors' main conclusion.

Version 2:

Reviewer comments:

Reviewer #1

(Remarks to the Author)
All comments are addressed.

Open Access This Peer Review File is licensed under a Creative Commons Attribution 4.0 International License, which permits use, sharing, adaptation, distribution and reproduction in any medium or format, as long as you give appropriate credit to the original author(s) and the source, provide a link to the Creative Commons license, and indicate if changes were made.
In cases where reviewers are anonymous, credit should be given to 'Anonymous Referee' and the source.
The images or other third party material in this Peer Review File are included in the article's Creative Commons license, unless indicated otherwise in a credit line to the material. If material is not included in the article's Creative Commons license and your intended use is not permitted by statutory regulation or exceeds the permitted use, you will need to obtain permission directly from the copyright holder.
To view a copy of this license, visit https://creativecommons.org/licenses/by/4.0/

REVIEWER COMMENTS

Reviewer #1 (Remarks to the Author):

The delivery of therapeutic cargo to resting primary T cells via nanoparticles poses significant challenges due to their inherently low transfection efficiency. In this study, Cevaal et al. suggest that targeting surface receptors with rapid internalization kinetics may enhance nanoparticle uptake and, consequently, improve transfection efficiency. Through in vitro internalization assay, the authors identified CD2 and CD7 as T cell surface markers with notably high internalization rates. They demonstrate that lipid nanoparticles (LNPs) functionalized with antibodies against CD2 or CD7 facilitate efficient mRNA delivery to primary T cells both in vitro and in vivo, utilizing humanized mouse models. The data presented are compelling, offering a promising strategy to address key challenges in T cell targeting. This work signifies a meaningful advancement in nanoparticle-mediated therapeutic delivery to primary T cells. However, the study also highlights several limitations, which are discussed below:

1. The intrinsic recycling rate of a surface marker can be significantly influenced by the binding epitope and affinity of the antibody that induces internalization. The current findings are derived from a single antibody clone chosen by the authors, raising questions about the generalizability of their conclusions to other antibody clones that target the same marker but possess different epitopes and affinities. For instance, although the authors assert that endocytosis for CD4 and CD5 is minimal, previous research has demonstrated that lipid nanoparticles (LNPs) functionalized with anti-CD4 or anti-CD5 antibodies can effectively mediate mRNA delivery to primary T cells in vivo. At the very least, for markers like CD2 and CD7, the authors should evaluate results across multiple antibody clones with clearly documented epitope specificity and binding affinities.

We thank the reviewer for their helpful comments and have repeated our analysis of receptor internalisation kinetics in primary $CD4^+$ T cells for CD4, CD2 and CD7 using two additional antibody clones for reach receptor target. These data are included in the revised manuscript in Supplementary Figure 5.

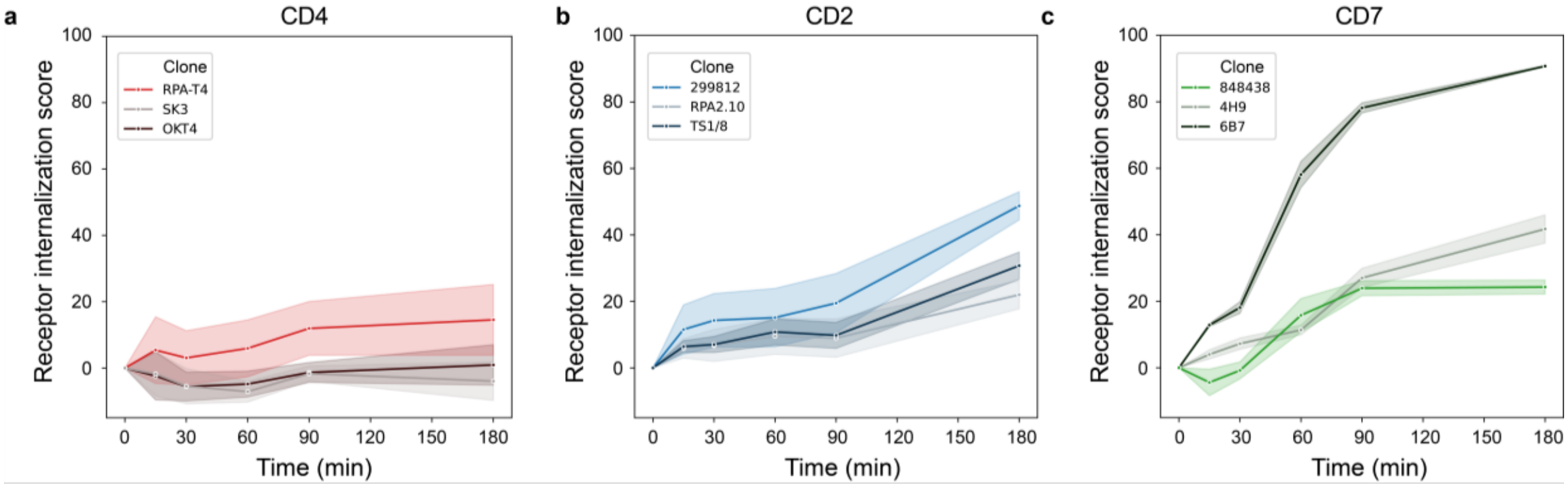

Figure S5. Receptor internalisation may be altered by some but not all antibody clones. Receptor internalisation score (IS) over time of CD4 (a), CD2 (b) and CD7 (c) receptors expressed on primary $CD4^+$ T cells from HIV-negative donors. The antibody clones tested initially in this manuscript are shown in red (CD4), blue (CD2) and green (CD7) shading; additional commercially available antibody clones against each receptor are shown in light and dark shades. n=4, values shown as mean ± SEM.

We found that with a non-internalising receptor such as CD4, using different antibody clones did not change the internalisation profile. For receptor targets that exhibited internalisation, we indeed saw some differences between antibody clones:

"Last, while the low and high internalisation observed for CD4 and CD7 respectively, corresponds closely to prior literature[20,21,38], we wanted to determine if the specific antibody clone had an impact on the receptor internalisation kinetics. For CD4, our prototype non-internalising receptor, we observed a low level of receptor internalisation using two additional anti-CD4 antibody clones (Figure S5a). For CD2 and CD7, we observed receptor internalisation regardless of the antibody clone used, although differences in receptor internalisation kinetics were observed with different clones (Figure S5b, c). In particular, anti-CD7 antibody clone 6B7 exceeded the speed and total degree of receptor internalisation observed with the original anti-CD7 clone 84838. The data suggests that in addition to the target itself, the antibody clone also has an impact on the internalisation kinetics.", lines 127-136

Details of the additional antibody clones used have been incorporated into the Methods:

"To assess the effect of antibody clones on receptor internalisation, the following additional antibodies were purchased: anti-CD2 (RPA2.10, 300242, Biolegend and TS1/8, 208242, Biolegend), anti-CD4 (OCT4, 566681, BD Biosciences and SK3, 244636, Biolegend), anti-CD7 (4H9, 568094, BD Biosciences and 6B7, 343118, Biolegend).", lines 311-315

Unfortunately, given we used commercially available antibodies, the exact binding epitopes and binding affinities of the available antibody clones were unknown. We have modified our discussion to make mention of this limitation:

"Last, while we found that receptor internalisation was determined by both the target and the antibody clone, the target epitopes and relative binding affinities of each clone were unknown. It remains possible that antibody clones targeting different epitopes induce different receptor internalisation patterns. The importance of the specific epitopes targeted therefore needs further study.", lines 290-295

2. The authors demonstrated that the internalization kinetics of the same surface markers, such as CD3 and CD5, vary significantly between Jurkat cells and primary CD4$^+$ T cells. This variation underscores the importance of cellular context in determining the internalization kinetics of these markers. To enhance the clinical relevance of these findings, it is essential to compare the internalization kinetics of CD2 and CD7 in primary CD4$^+$ T cells derived from healthy donors with those from patients.

We have now performed these experiments as requested and analysed the receptor internalisation for CD2, CD7 and CD4 in CD4$^+$ T cells from people with HIV on antiretroviral therapy (ART) The details and ethics of obtaining samples from people with HIV has been included in the Methods:

"Blood samples from people with HIV were collected through leukapheresis at the University of California San Francisco (UCSF) (USA) with informed consent and under institutional guidelines. People with HIV included in these studies were aged

18 years or older and on suppressive antiretroviral treatment (ART) (viral load < 50 copies/mL) for >3 years. The use of these samples was approved by the Human Research Ethics Committees at the University of Melbourne and the Institutional Review Board at UCSF.". lines 325-330

These findings are incorporated into the revised manuscript as Supplementary Figure 4:

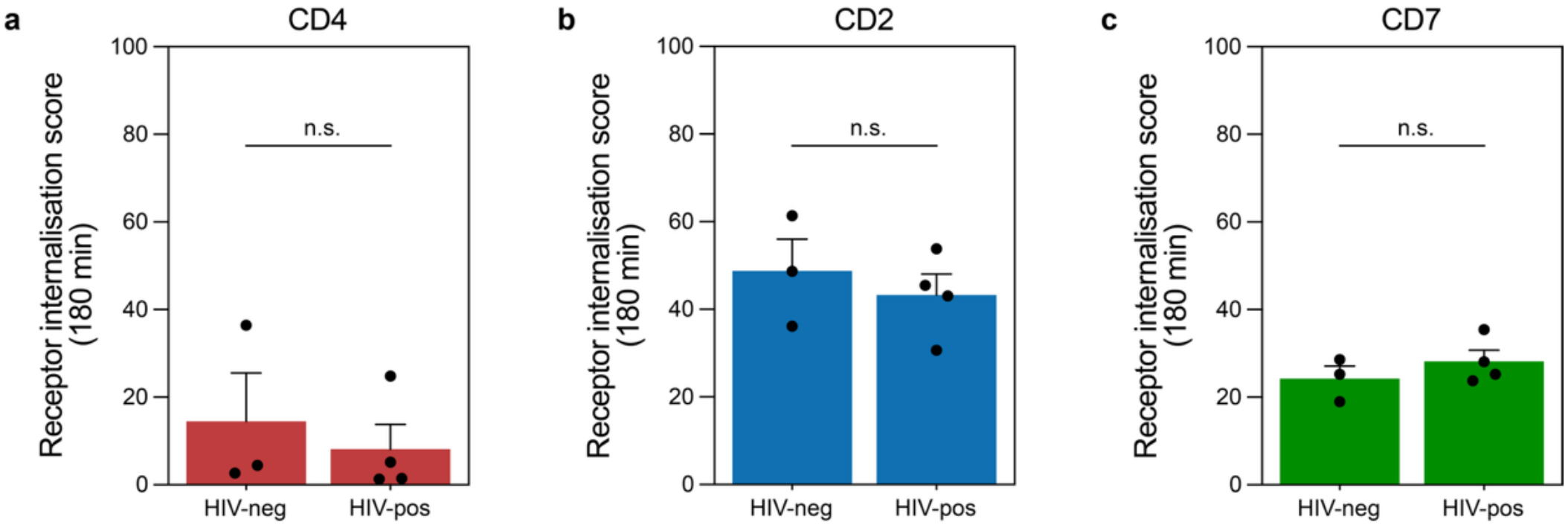

Figure S4. Degrees of receptor internalisation are similar in CD4⁺ T cells isolated from HIV-negative donors and people with HIV on ART. Receptor internalisation score (IS) after 180 min of CD4 (a), CD2 (b) and CD7 (c) receptors expressed on primary CD4⁺ T cells from HIV-negative donors or people with HIV on ART. n=3-4, values shown as mean ± SEM. Each donor is shown as a solid circle. Significance was determined through two-way unpaired t-test; n.s. non-significant, $p > 0.05$.

We found no significant difference in receptor internalisation using CD4⁺ T cells from HIV-negative donors or people with HIV on ART:

"Furthermore, receptor internalisation did not significantly differ between CD4⁺ T cells from people with HIV on ART and CD4⁺ T cells from HIV-negative donors (Figure S4).", lines 124-126

We make a further reference to these findings in the Discussion:

"CD2 and CD7 receptor internalisation was maintained in CD4⁺ T cells from people with HIV, demonstrating the clinical relevance of our findings.", lines 258-259

3. Characterization of the density of CD2 and CD7 antibodies on the functionalized, mRNA-loaded lipid nanoparticles is missing.

mRNA-loaded lipid nanoparticles were functionalised with CD2 or CD7 antibodies following a protocol that has now been published in Nature Nanotechnology (previously only available on BioRXiv). This study characterised the density of antibody-capturing nanobodies on the lipid nanoparticle surface and found a density of ~200 nanobodies per particle. Furthermore, the authors developed an optimized protocol for subsequent addition of the targeting antibody, in which the antibody is added at a two-fold excess of nanobody to antibody. Under these conditions, all antibodies are captured, resulting in an estimated antibody density of ~100 antibodies per particle. Please see:

Chen, M. Z. *et al.* A versatile antibody capture system drives specific in vivo delivery of mRNA-loaded lipid nanoparticles. *Nat Nanotechnol* **20**, 1273–1284 (2025). https://doi.org:10.1038/s41565-025-01954-9

We have updated our referencing and added a specific mention of this characteristic in the Methods section:

"Nanobody-DSPE-PEG2000 conjugates were post-inserted into the surface of the LNPs, resulting in a nanobody array with the Fc-binding site facing outward at a density of ~200 nanobodies per LNP[44]. (…) Targeting antibodies were added at a two-fold excess of nanobody to antibody to ensure efficient capture of all antibodies, resulting in an estimated density of ~100 antibodies per LNP[44].", line 435-443

4. Since the in vitro receptor internalization assays for CD2 and CD7 were conducted in CD4⁺ T cells rather than CD8⁺ T cells, it would be more accurate to revise the title by replacing "primary T cells" with "primary CD4⁺ T cells."

This has now been revised as per the reviewer's suggestion.

5. Previous studies have shown that LNPs functionalized with anti-CD3 or anti-CD5 antibodies can effectively deliver mRNA to primary T cells in vivo, which seems to contrast with the conclusions presented in this manuscript. The authors should address these discrepancies in the discussion by comparing their findings with earlier research and providing possible explanations for the observed inconsistencies.

We thank the reviewer for highlighting this, but we believe that our findings are consistent with earlier studies. CD3 internalisation has been well-characterised as part of the T cell receptor (TCR)/CD3 complex. Our findings indeed confirmed that CD3 internalises upon antibody binding in primary $CD4^+$ T cells (Figure 1) and CD3-targeted nanoparticles were internalised in primary $CD4^+$ T cells (Figure 2).

In our hands, CD4 and CD5 exhibited minimal internalisation in primary $CD4^+$ T cells (Figure 1). These findings are not contradictory to the earlier studies showing that CD4- or CD5-targeted nanoparticles outperform non-targeted nanoparticles. We acknowledge these studies in the Discussion; however, we hypothesise that nanoparticle uptake and delivery of cargo can be improved upon further (by targeting a rapidly-cycling receptor):

"CD4-targeted lipid nanoparticles have previously been shown to enhance mRNA delivery to $CD4^+$ T cells *in vitro* and *in vivo* compared to untargeted control particles[17]. Yet, despite being specifically designed to target T cells, in this study less than 1% of particles added associated with T cells[17], indicating a clear need for improved targeting approaches.", lines 268-271

Even so, as we show in the Figure S5, the antibody selected for targeting can impact the efficiency of receptor internalisation. We tested three clones to CD4 and none exhibited high receptor internalisation kinetics. We acknowledge the possibility that other clones, such as clone A161A1 (described in Tombacz et al Mol Ther 2021) may have more favourable kinetics. We now include a comment on this in the Discussion, which also addresses Reviewer comment #1 and the newly added Figure S5:

“Last, while we found that receptor internalisation was determined by both the target and the antibody clone, the target epitopes and relative binding affinities of each clone were unknown. It remains possible that antibody clones targeting different epitopes induce different receptor internalisation kinetics. The importance of the specific epitopes targeted therefore needs further study.”, lines 290-295

Reviewer #1 (Remarks to the Author):

Although some questions are addressed, but there are a couple of concerns with the data provided. For question #1, the authors followed our suggestions and tested three different antibody clones. There are two concerns with the data (fig. S5)

1. In the original report, aCD7 (clone 84838) showed an internalization score of 61.8 ± 4.0 in CD4⁺T cells after 180 minutes (Fig.1 K-l). However, in this subsequent repeat experiment comparing three different aCD7 clones, the same clone (84838) exhibited an internalization score of less than 20 at the same time point (Fig.S5). This substantial inter-experimental variability (≈62 vs <20) warrants further explanations.

We had indeed noted the discrepancy in relation to clone 848438 the reviewer highlights - between the original and revised manuscripts. We decided to include the data, as the new experiments were designed to address a separate research question that required comparisons within datasets i.e. comparisons between clones or comparisons between HIV-positive and HIV-negative samples.

Given the reviewers' concerns about reproducibility of findings with clone 848438 between the original submission (R0) and first resubmission (R1), we have performed multiple additional experiments to investigate this discrepancy in detail and submit our revised findings in this second resubmission (R2).

We have now performed a further <u>three repeat independent experiments (using different donors in each experiment)</u> and can confirm that the data in the original manuscript was correct. The repeat experiments with clone 848438 resulted in a consistently high CD7 internalisation score, with the mean of experimental repeats ± SEM being 63.4 ± 4.4 after 180 min – consistent with the data presented in the original manuscript (R0, shown originally in Figure 1k-l, and with a CD7 internalisation score of 61.8 ± 4.0 at 180 minutes). All experiments performed using clone 848438 for R0, R1 and R2 are summarised in figure 1 and table 1 below.

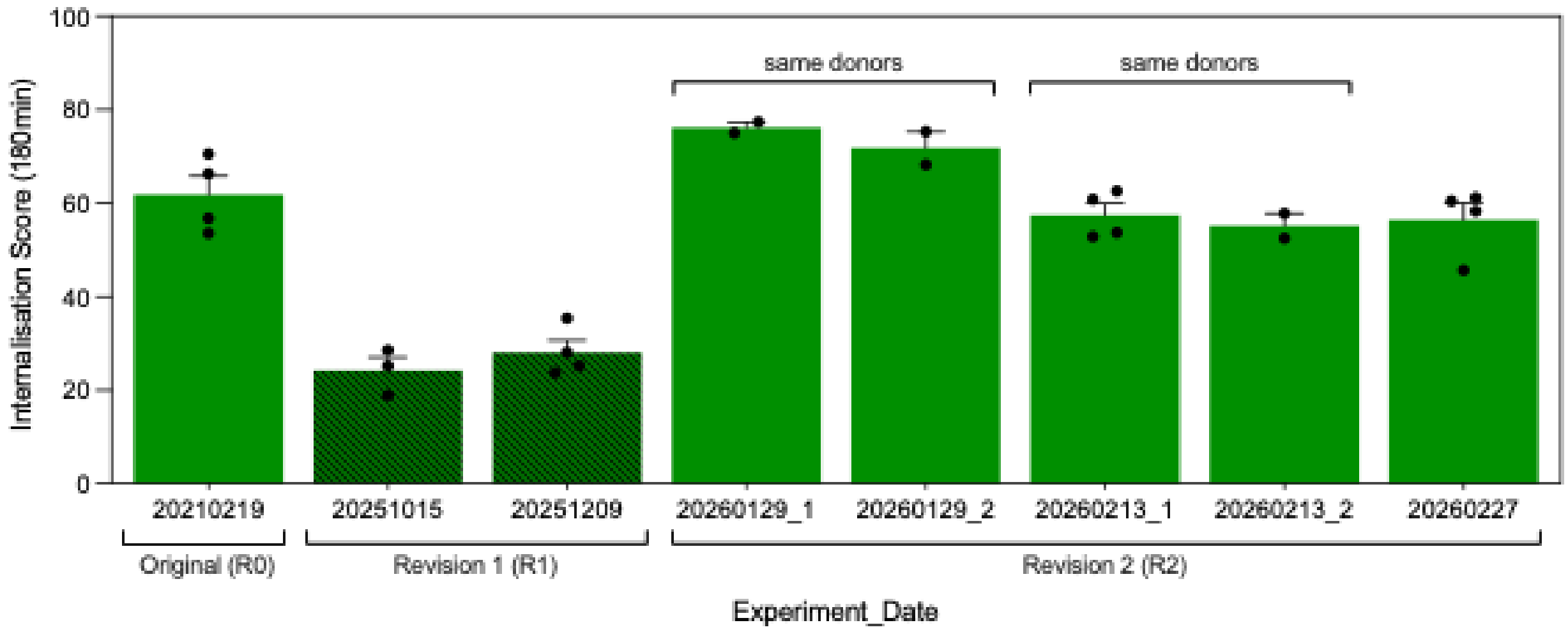

Figure 1: Receptor internalisation assay for anti-CD7 performed in the original and revised manuscripts. Comparison of receptor internalisation scores (IS) after 180min using anti-CD7 antibody clone 848438, as presented previously in Fig 1k-l (original manuscript, R0), Fig S4 and Fig S5 (revision 1, R1), as well as five subsequent repeat experiments (revision 2, R2). Each solid black circle represents a different donor within the one experiment. The height of the column is the mean and error bars SEM.

Table 1: Data from experiments for receptor internalisation assay for anti-CD7 and clone 848438. R0 = original submission, R1 = first resubmission and R2 = second resubmission. The dates of each experiment are also shown.

	Date	Number of donors	Internalisation score (180min) (mean)	Internalisation score (SEM)	%CD7+	%viability
**R0**	20210219	4	61.8	4.0	88.6	93.4
**R1**	20251015	3	24.3	2.8	90.2	96.7
	20251209	4	28.2	2.6	90.0	95.0
**R2**	20260129	2	71.8	3.5	93.8	97.5
	20260129	2	76.2	1.2	93.7	97.2
	20260213	4	57.5	2.9	88.3	96.7
	20260213	2	55.2	2.7	92.1	96.9
	20260227	4	56.4	3.6	86.2	94.8

Given we observed lower CD7 internalisation in the first revision (R1) compared to the original submitted data (R0) and the recently repeated data (R2), we next investigated potential causes for the results from R1:

b. Donor effect – given that the experiments in R0, R1 and R2 were performed in different sets of donors, it is possible that a donor effect caused the lower internalisation scores in R1. However, as the results within R1 were consistent between the n=7 independent donors tested (as part of experiments 20251015 and 20251209, see table 1) and again consistent between the n=10 donors screened as part of R2, we deem this possibility unlikely. Furthermore, there was no significant difference in CD2 or CD4 receptor internalisation score between R0 and R1, isolating the issue to CD7 (figure 2 below).

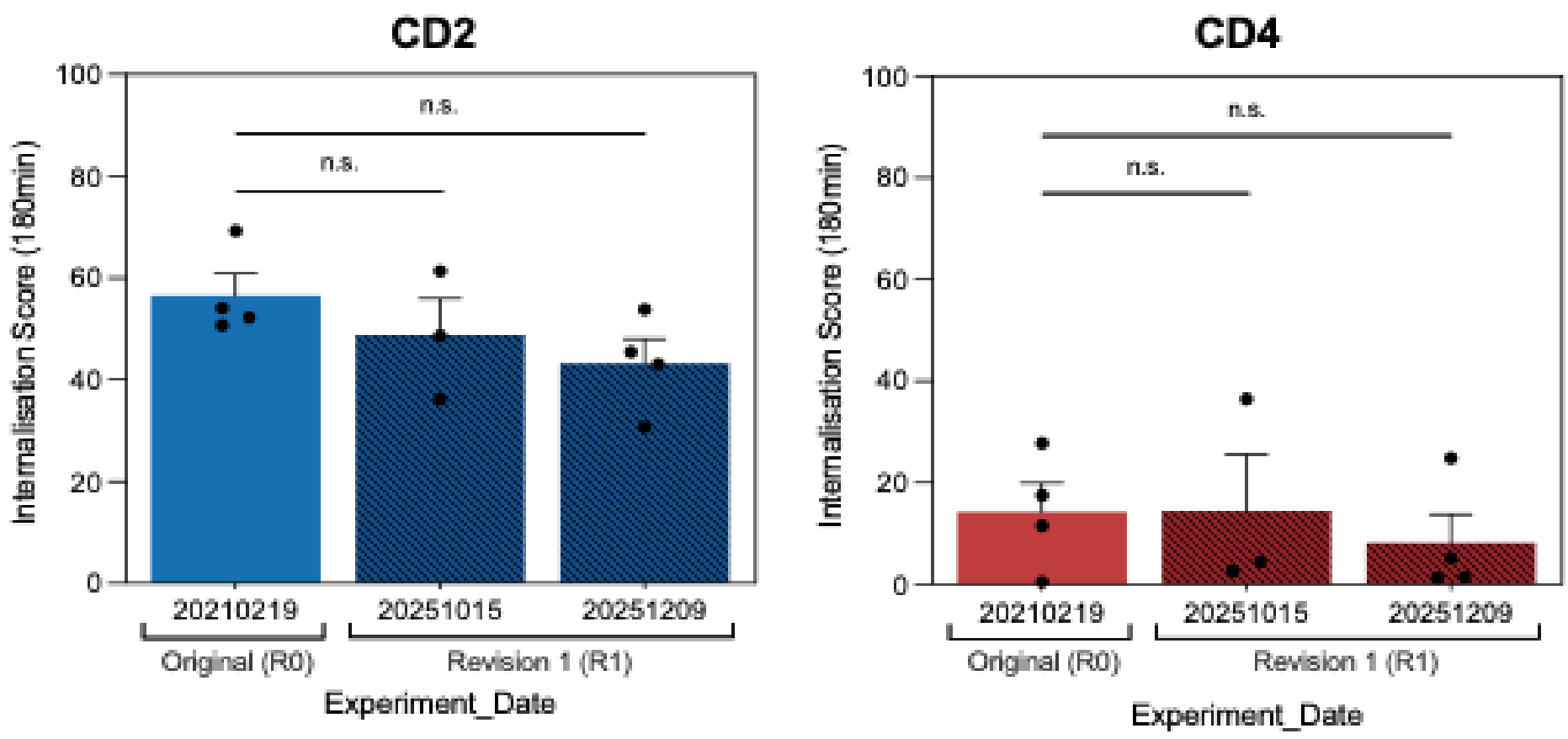

Figure 2: Receptor internalisation scores for anti-CD2 and anti-CD7 in separate experiments. Receptor internalisation scores (IS) after 180min using anti-CD2 antibody clone 299812 (blue) and anti-CD4 antibody clone RPA-T4 (red) showed no significant differences between the original submission (R0) and first resubmission (R1). Significance was determined using two-way unpaired t-test; n.s. non-significant, $p > 0.05$.

c. Difference in cell viability – we hypothesised that differences in the viability of the primary CD4+ T cells could result in lower receptor internalisation, however, no differences in cell viability were observed between R0, R1 and R2 in CD7-stained conditions, with viabilities > 90% across all experimental repeats (see table 1).

d. Differences in CD7 primary antibody staining efficiency – we found no difference in the percentage of CD7+ cells, consistent with similar levels of staining with the primary antibody (see table 1).

e. Operator differences – all experimental repeats (R0, R1 and R2) were performed by the same operator and likewise, the analysis of all experimental repeats was performed by the same operator.

f. Analysis – we reviewed the flow cytometry gating strategies for R0, R1 and R2 and found no difference. Downstream analysis and calculations of receptor internalisation scores were performed using the same, automated Python script.

g. Preparation of primary CD7 antibody (clone 848438) – we hypothesised that inadvertent incomplete buffer exchange of the primary CD7 antibody during R1 could result in residual presence of sodium azide, which is a known inhibitor of endocytosis. We performed a direct comparison between antibody solution used for R1 with a freshly buffer-exchanged antibody preparation (used for R2). We found no difference between the old and new antibody solution, with both datasets showing high CD7 internalisation (figure 3).

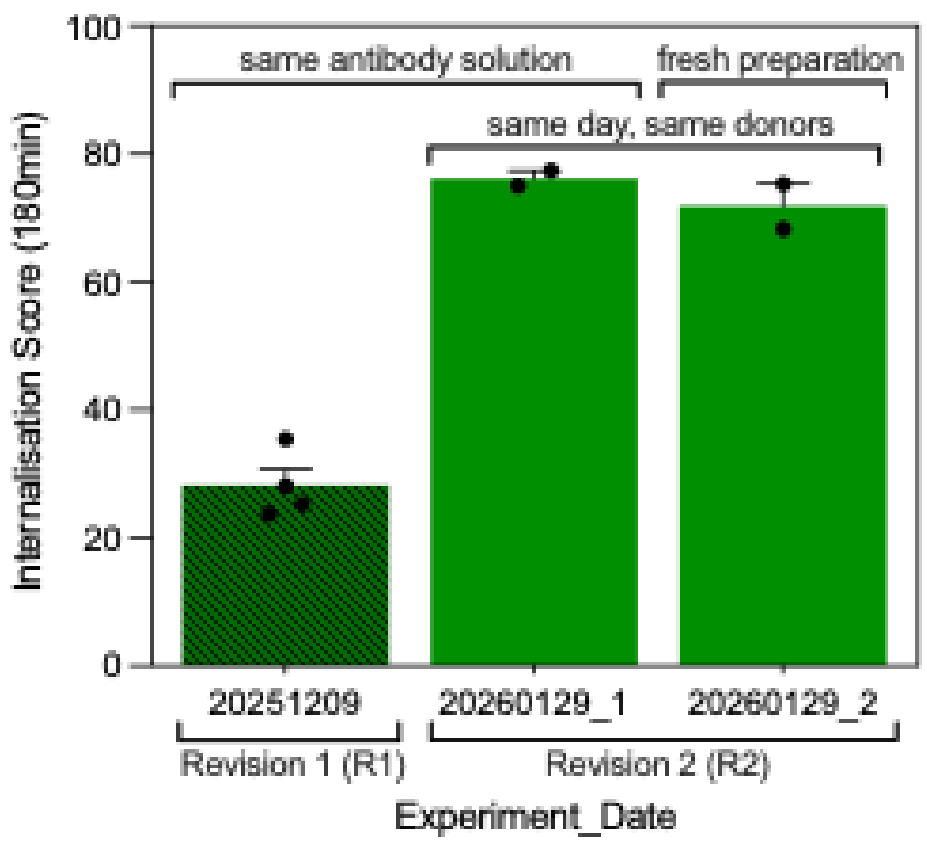

Figure 3: Lower receptor internalisation score in R1 is not linked to the CD7 antibody preparation. Receptor internalisation scores (IS) after 180min using anti-CD7 antibody clone 848438 were repeated in a single experiment, comparing internalisation when using leftover antibody solution as used in R1 (20260129_1) versus a fresh antibody preparation (20260129_2).

Upon broader comparison of R0, R1 and R2 data beyond CD7 clone 848438, we identified that CD7 clone 4H9 also exhibited lower receptor internalisation during R1 compared to R2, suggesting the discrepancy was not isolated to CD7 clone 848438 but was isolated to CD7 (figure 4). No significant difference was seen with clone 6B7, although the very high levels of internalisation seen with 6B7 may have obscured any differences.

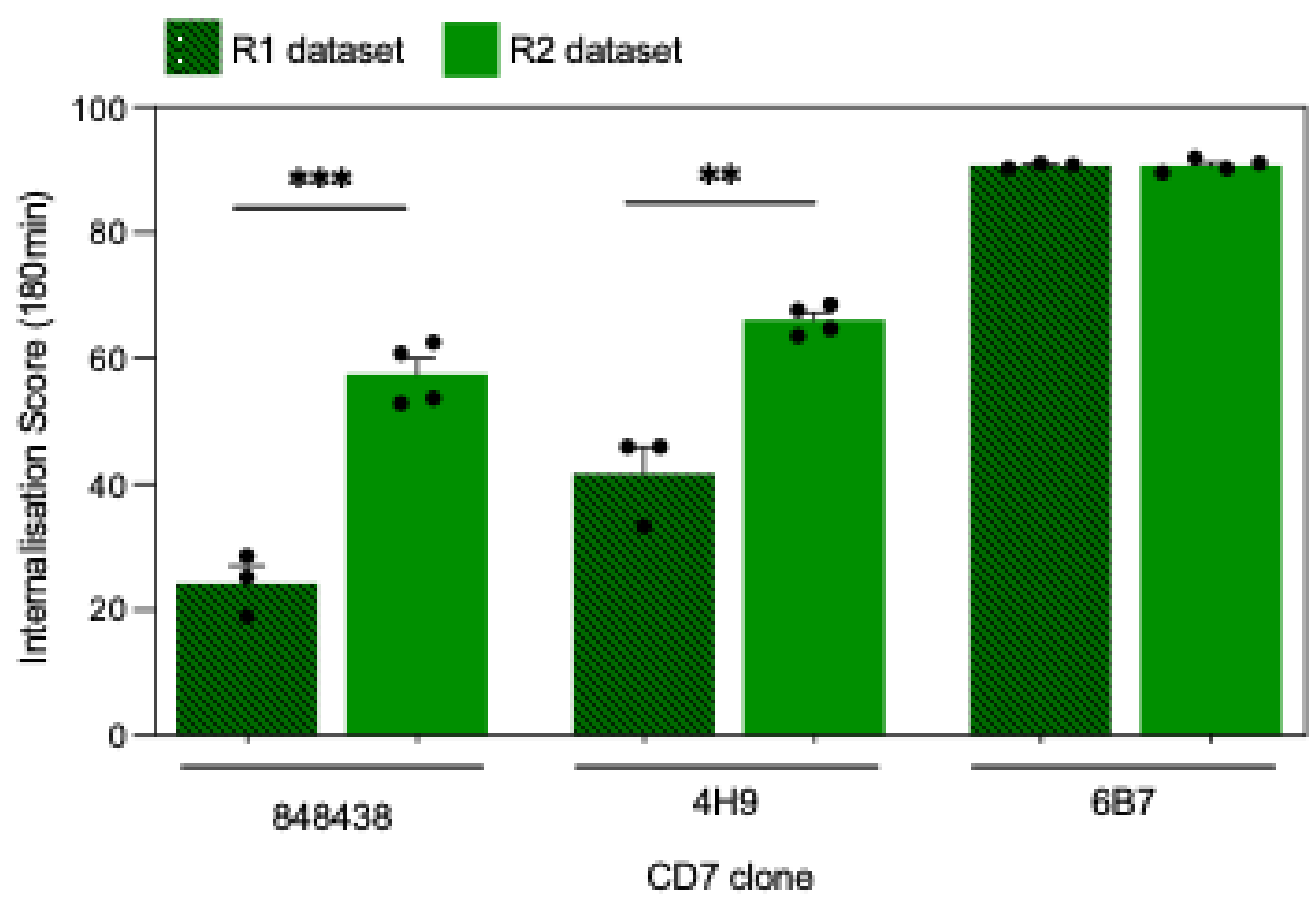

Figure 4: CD7 internalisation scores were lower in R1 compared to R2 for two anti-CD7 antibodies: clone 848438 and 4H9. Receptor internalisation scores (IS) after 180min using anti-CD7 antibody clone 848438, 4H9 and 6B7 as determined as part of R1 and R2 experimental datasets. Significance was determined using two-way unpaired t-test; *, $p < 0.05$; **, $p < 0.01$; ***, $p < 0.001$.

In summary, while we were unable to identify the cause of the lower internalisation scores for R1, our multiple new repeat experiments of CD7 internalisation (now in 10 donors, including both HIV negative and HIV positive donors as well) are consistent with our original data set (R0) and consistent with our *in vitro* and *in vivo* data using CD7-targeted nanoparticles (Figure 2-5 in the manuscript). Based on this we believe that the data from R1 was incorrect.

Using data from R0 and R2 (total of 6 experiments on 14 donors), we have now calculated the intra-assay coefficient of variation (CV) as *%CV = SD of replicate mean ÷ replicate mean × 100* and the inter-assay CV as *%CV = SD of experiment means ÷ mean of experiment means × 100*. Our assay demonstrates an intra-assay CV of 8.38% and an inter-assay CV of 11.16% which we believe supports the robustness of our assay.

We have updated Figure S4c and Figure S5c of the manuscript with data from these new experiments. Importantly, the conclusions from these Supplementary Figures remain unchanged; therefore, no further changes to the text manuscript were made.

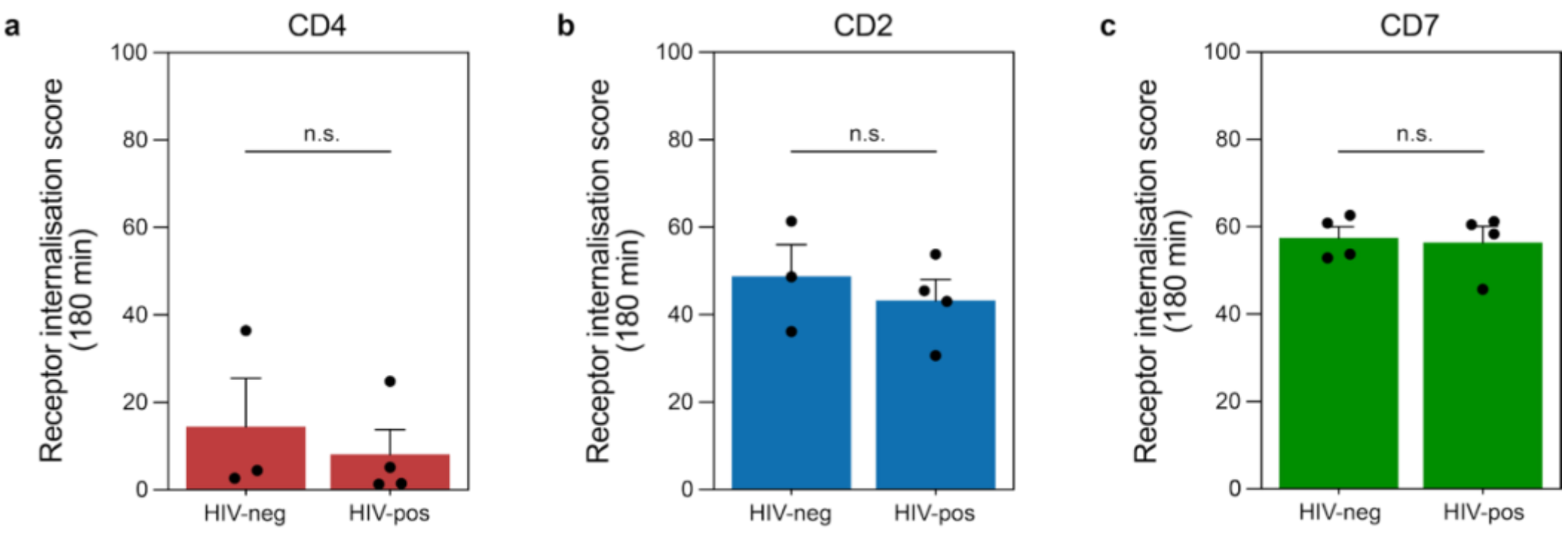

Figure S4. Degrees of receptor internalisation are similar in CD4⁺ T cells isolated from HIV-negative donors and people with HIV on ART. Receptor internalisation score (IS) after 180 min of CD4 (a), CD2 (b) and CD7 (c) receptors expressed on primary CD4⁺ T cells from HIV-negative donors or people with HIV on ART. n=3-4, values shown as mean ± SEM. Each donor is shown as a solid circle. Significance was determined through two-way unpaired t-test; n.s. non-significant, $p > 0.05$.

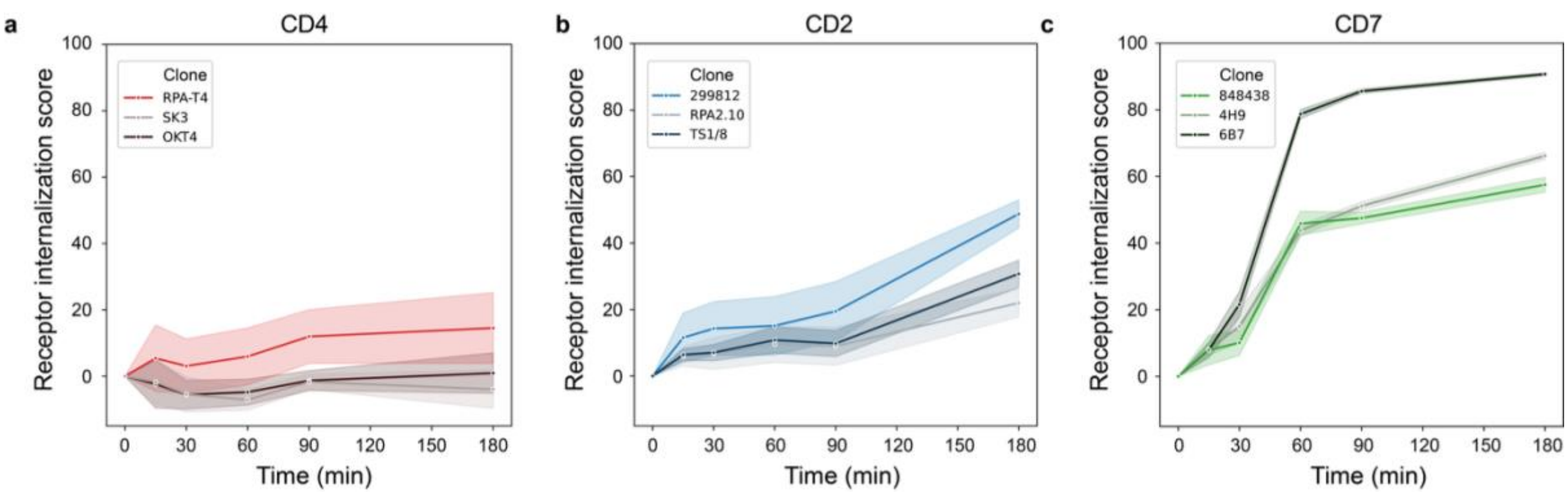

Figure S5. Receptor internalisation may be altered by some but not all antibody clones. Receptor internalisation score (IS) over time of CD4 (a), CD2 (b) and CD7 (c) receptors expressed on primary CD4⁺ T cells from HIV-negative donors. The antibody clones tested initially in this manuscript are shown in red (CD4), blue (CD2) and green (CD7) shading; additional commercially available antibody clones against each receptor are shown in light and dark shades. n=4, values shown as mean ± SEM.

2. The internalization score of the aCD7 clone 6B7 is approximately twofold higher than that of clone 4H9 and fourfold higher than that of the original clone 84838 (Fig.S5). This magnitude of clone-dependent variability raises the question of whether nanoparticle delivery efficiency to CD4 ⁺T cells is driven primarily by intrinsic CD7 receptor internalization kinetics or by the specific antibody clone used for targeting. Additional evidence is needed to distinguish between these two possibilities in order to endorse the authors' main conclusion.

To explain the differences in CD7 internalisation observed between antibody clones, we hypothesised that clone 6B7 may trigger cellular activation, which would drive a greater degree of receptor internalisation. Our data, however, do not support this hypothesis, as we found that none of the anti-CD7 antibody clones induced activation marker expression on primary CD4 T cells. We have added this data as additional panels (d-f) in Figure S5, and added the following changes in-text:

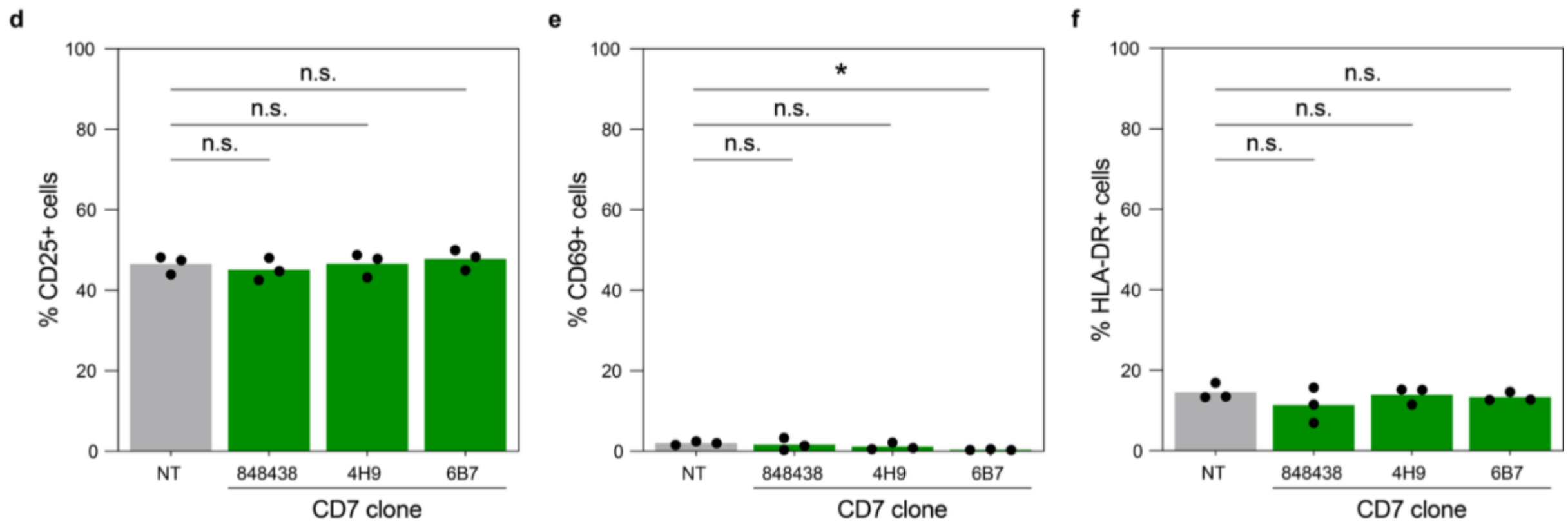

Figure S5. Receptor internalisation may be altered by some but not all antibody clones. [...] (d-f) Percentage of primary CD4$^+$ T cells expressing activation markers CD25 (d), CD69 (e) and HLA-DR (f) after 48 hr upon staining with original (848438) and additional anti-CD7 antibody clones (4H9 and 6B7), compared to non-treated (NT) control cells not incubated with any antibody. n=3, values shown as mean with individual datapoints. Significance was determined through RM one-way ANOVA; * $p < 0.05$, n.s. $p > 0.05$

"In particular, anti-CD7 antibody clone 6B7 exceeded the speed and total degree of receptor internalisation observed with the original anti-CD7 clone 848438.This difference could not be explained by differences in the induction of T cell activation markers between the anti-CD7 antibody clones (Figure S5d-f). Combined, these data suggest that both the intrinsic target and the antibody clone can dictate internalisation.", lines 133 – 137

Details of this additional experiment have been included in the Methods:

"To assess the induction of T cell activation markers by the different anti-CD7 antibody clones, primary total CD4$^+$ T cells were incubated with primary antibody as above and excess antibody removed. Cells were then incubated for 48 hr prior to staining against HLA-DR (347363, BD Biosciences), CD69 (564364, BD Biosciences) and CD25 (356108, Biolegend).", lines 354 – 357

We have updated our Discussion to further address this point. A detailed study comparing target epitopes and binding affinities is not possible with the commercially obtained antibody clones used in this study and is therefore beyond the scope of this work.

“Last, we found that for the rapidly-internalising receptor CD7, receptor internalisation was further enhanced using one particular antibody clone (6B7). As the target epitopes and relative binding affinities of each clone are unknown, it is plausible that antibody clones targeting different epitopes induce different receptor internalisation kinetics. The importance of the targeted receptor epitope therefore needs further study.”, lines 291 – 296
